# Carbon Nanostructures—Silica Aerogel Composites for Adsorption of Organic Pollutants

**DOI:** 10.3390/toxics11030232

**Published:** 2023-02-28

**Authors:** Alyne Lamy-Mendes, David Lopes, Ana V. Girão, Rui F. Silva, Wim J. Malfait, Luísa Durães

**Affiliations:** 1 University of Coimbra, CIEPQPF—Chemical Process Engineering and Forest Products Research Centre, Department of Chemical Engineering, 3030-790 Coimbra, Portugal; 2 CICECO—Aveiro Institute of Materials, Department of Materials and Ceramic Engineering, University of Aveiro, 3810-193 Aveiro, Portugal; 3 Laboratory for Building Energy Materials and Components, Empa—Swiss Federal Laboratory for Science and Technology, Überlandstrasse 129, 8600 Dübendorf, Switzerland

**Keywords:** silica-based aerogels, carbon nanotubes, graphene oxide, adsorption

## Abstract

Silica aerogels are a class of materials that can be tailored in terms of their final properties and surface chemistry. They can be synthesized with specific features to be used as adsorbents, resulting in improved performance for wastewater pollutants’ removal. The purpose of this research was to investigate the effect of amino functionalization and the addition of carbon nanostructures to silica aerogels made from methyltrimethoxysilane (MTMS) on their removal capacities for various contaminants in aqueous solutions. The MTMS-based aerogels successfully removed various organic compounds and drugs, achieving adsorption capacities of 170 mg⋅g^−1^ for toluene and 200 mg⋅g^−1^ for xylene. For initial concentrations up to 50 mg⋅L^−1^, removals greater than 71% were obtained for amoxicillin, and superior to 96% for naproxen. The addition of a co-precursor containing amine groups and/or carbon nanomaterials was proven to be a valuable tool in the development of new adsorbents by altering the aerogels’ properties and enhancing their adsorption capacities. Therefore, this work demonstrates the potential of these materials as an alternative to industrial sorbents due to their high and fast removal efficiency, less than 60 min for the organic compounds, towards different types of pollutants.

## 1. Introduction

A significant increase in aquatic pollution has been observed due to the expansion of industrial complexes. Notable amounts of organic and inorganic pollutants are released with wastewater, leading to the contamination of aquatic environments such as natural aquifers [1,2]. Aromatic compounds are still largely used in the petrochemicals’ industry for extracting and producing in-demand goods, and also are found in petroleum and gasoline [3,4,5], however, because of their toxicity, which, in some cases, has been linked to carcinogenic and mutagenic effects, there is an increasing necessity to remove these contaminants from effluents [2,6,7]. Some of the volatile organic compounds (VOCs) are extremely toxic even at low concentrations, as in the case of phenol [8], and long-term exposure to benzene and xylene, for example, has a variety of negative effects on human health, such as liver lesions and cancer [9].

Besides these well-known contaminants, pharmaceutical active compounds (PhACs) have been recently identified as emerging pollutants, and, due to the growth in their consumption, they are continuously entering the environmental media, particularly aquatic ones, due to the effluent from industrial, urban, or hospital wastewater treatment plants [10,11,12]. From the 150 pharmaceuticals used daily in significant amounts worldwide, 55 of them are detected in relevant concentrations [13,14,15]. Amoxicillin (AMX), a broad-spectrum β-lactam antibiotic, is one of the most widely produced drugs and most commonly used antibiotics [16], while Naproxen (NPX) is a non-steroidal anti-inflammatory drug, being one of the most prescribed among this category [17]. As a consequence, both drugs have been detected in water resources [18,19,20,21,22] and wastewater sewers [23,24,25,26,27]. The presence of these PhACs can lead to the thriving and expansion of antibiotic resistance genes (ARGs), as even nonantibiotic pharmaceuticals, including NPX, contribute to the horizontal transfer of ARGs [28] and the lower effectiveness in treating the original diseases [29]. Moreover, as these PhACs are designed to induce a physiological response, their presence in the aquatic environment is problematic since they can potentially affect both aquatic and human lives [10,30].

Due to the increasing difficulties in complying with regulations, more traditional wastewater treatments must be improved [31]. Among the various technologies available to remove pollutants from water environments, the adsorption process has piqued a lot of interest [32,33], as it is an effective and simple methodology that can be applied for a broad range of contaminants [34,35]. However, due to their low adsorption capacities, poor removal efficiency, and slow kinetics [36,37], common industrial sorbents such as clays, alumina, mesoporous SiO_2_ or zeolites [8,38,39] are limited in their use.

Silica aerogels are a viable alternative for circumventing these constraints. The possibility of altering their surface chemistry by changing their functional groups or charge, combined with their high porosity, makes them viable adsorbents [36,40,41]. A few studies have already been performed to assess the impact of these surface modifications on the silica aerogels’ adsorption performance. Štandeker et al. [42] altered the degree of hydrophobicity of the aerogels by adding methyltrimethoxysilane (MTMS) or trimethylethoxysilane (TMES) in tetramethoxysilane (TMOS)-based aerogels. These modifications led to adsorption capacities 15 to 400 times higher than the ones obtained by granulated active carbon for pollutants such as benzene, toluene, chloroform, and chlorobenzene. Sun et al. [43] also observed that hydrophilizing sodium silicate–silica aerogel microspheres by methyl grafting had a positive impact on the sorption capacities for pump oil and hexane, reaching values of 17.9 g⋅g^−1^ and 11.8 g⋅g^−1^, respectively. High removal rates of phenol from water were achieved by Qin et al. [44] when the hydrophilic character of tetraethyl orthosilicate (TEOS)-based aerogels was altered by the introduction of trimethylchlorosilane (TMCS). When the equilibrium concentration of phenol was 290 mg⋅L^−1^, the authors reached a maximum adsorption capacity of 142 mg⋅g^−1^ with the modified aerogels.

According to the research shown above, the interaction between silica aerogels and pollutants can be enhanced by altering the surface chemistry of the aerogels. In addition to the presence of methyl groups, the inclusion of amino and carboxyl functional groups can also significantly alter their compatibility with specific contaminants [45,46]. Another possible modification that can be made in the silica aerogels, to further improve their adsorption capacities, is the addition of carbon nanostructures, which are highly efficient adsorbents for water treatments due to their various morphologies and high specific surface area [47]. Moreover, these materials also allow chemical modification and functionalization [47,48], which can enhance their affinity with emerging chemical contaminants.

Given the improved affinity and interaction of amino groups and carbon nanomaterials with a variety of pollutants, the aim of this work is to show how the adsorption capacity, for different contaminants in aqueous solutions, of MTMS-based silica aerogels is affected by an amino functionalization and/or the addition of carbon nanotubes or graphene oxide. To the best of our knowledge, the use of carbon nanomaterials-MTMS-based silica aerogel composites as adsorbents has not yet been reported. Moreover, based on promising results from the previous work of our group [49], the effect of amino functionalization, combined with the addition of carbon nanostructures, on the removal efficiency of different pollutants was assessed. The chosen pollutants, benzene, toluene, xylene, phenol, amoxicillin, and naproxen, have high environmental relevance owing to their toxicity and persistency and are also commonly found in different wastewaters.

## 2. Materials and Methods

### 2.1. Materials

Methyltrimethoxysilane (MTMS; purity ≥ 98%; Aldrich, Milwaukee, WI, USA; CH_3_Si(OCH_3_)_3_) and (3-aminopropyl)trimethoxysilane (APTMS; purity ≥ 97%; Aldrich; H_2_N(CH_2_)_3_Si(OCH_3_)_3_) were the silica precursor and co-precursor, respectively. As solvents, ethanol (absolute, Fluka; C_2_H_5_OH) and distilled water were used. Oxalic acid anhydrous (purity ≥ 99%, Fluka; C_2_H_2_O_4_) was used as the acid catalyst, while ammonium hydroxide (25% NH_3_ in H_2_O; Fluka Analytical, Buchs, Switzerland; NH_4_OH) was used as the basic catalyst. Two surfactants were employed, hexadecyltrimethylammonium bromide (CTAB; purity ≥ 99%; Sigma, St. Louis, MO, USA; C_19_H_42_BrN) and poly(ethylene glycol) 600 (PEG; purity ≥ 99%; Sigma; H(OCH_2_CH_2_)nOH). The utilized carbon nanomaterials were commercial multi-walled carbon nanotubes (CNTs; purity 90%; Nanocyl, Sambreville, Belgium; average diameter of 9.5 nm, average length of 1.5 μm, surface area of 250–300 m^2^⋅g^−1^), and graphene oxide (GO; Graphenea, San Sebastián, Spain; concentration 0.4 wt.%, monolayer content (at 0.05 wt.%), ≥95%). The CNTs were modified with tetramethyl orthosilicate (TMOS; purity ≥ 99%; Aldrich; Si(OCH_3_)_4_) and nitric acid (purity 70%; Sigma Aldrich; HNO_3_). Pollutants’ solutions were prepared using benzene (purity ≥ 99%; Sigma Aldrich; C_6_H_6_), toluene (purity ≥ 99.5%; Sigma Aldrich; C_6_H_5_CH_3_), xylene (mixture of isomers; Sigma Aldrich; C_8_H_10_), phenol (purity ≥ 99%; Sigma Aldrich; C_6_H_5_OH), amoxicillin (AMX, 95.0–102.0% anhydrous basis; Sigma Aldrich; C_16_H_19_N_3_O_5_S) and naproxen (NPX; Sigma Aldrich; CH_3_OC_10_H_6_CH(CH_3_)CO_2_H). All reagents were used without prior purification. 

### 2.2. Synthesis of Carbon Nanomaterial-Silica Aerogel Composites

Initially, two amounts of surfactants were tested, 0.83 wt.% and 4.0 wt.%, for the silica aerogels. The dispersion of carbon nanostructures in the silica sol was critical to obtaining homogeneous samples, and, to accomplish this goal, the addition of a surfactant was required. CTAB was tested as a surfactant for all aerogel composites, but when it was added to the solution containing the solvent mixture and GO, a phase separation was observed. As a result, it was necessary to use a different type of surfactant; for the composites with CNTs, CTAB was used, while for the GO-silica aerogel composites, the surfactant was PEG.

The silica materials tested as adsorbents in this paper were prepared as described in previous works [50,51]. The composite materials were synthesized using a two-step acid-base catalyzed sol-gel process with oxalic acid (0.01 M) as an acid catalyst and ammonium hydroxide (1 M) as a basic catalyst. Ethanol and water were chosen as solvents, and MTMS and APTMS were used as the silica precursor and co-precursor, respectively.

The synthesis procedure starts with the surfactant and MTMS being added to the solvent mixture. After that, the acid catalyst was added to promote the hydrolysis reactions. The solution then received the addition of APTMS and ammonium hydroxide, which trigger gelation. Gelation and ageing steps were carried out at 27 °C, and the samples were aged for 7 days. Surfactant removal was accomplished through diffusional ethanol washing at 60 °C. The aerogel composites were dried for three days at ambient pressure at 60 °C and then for three hours at 100 °C.

The synthesized materials were designated as xMyA, where x in xM is the mol percentage of Si from MTMS and y in yA corresponds to the mol percentage of Si from APTMS. Two types of carbon nanotubes were here added to the silica matrix, CNT-HNO_3_ and CNT-TMOS, with the modification procedure being described in a previous work [50]. Briefly, the surface of CNTs was modified by refluxing them with concentrated HNO_3_ for 20 h at 50 °C, followed by washing, filtering, and drying. These CNTs were called CNTs-HNO_3_. After the treatment, carboxylic groups were observed in the CNT-HNO_3_ [50]. 

The silane treatment was the second modification step, in which the CNT-HNO_3_ were refluxed with a 10% TMOS solution for 4 h at 70 °C before being filtered and dried. These CNTs were labelled CNTs-TMOS. The effectiveness of the modification was confirmed by the presence of several silica-related bands in the FTIR spectrum and by the presence of silica around the CNTs, as reported in Ref. [50].

For the composites containing the carbon-nanomaterials, the designation xMyA_CNT_z or xMyA_GO_z was used, where z is the amount in mg of carbon nanomaterial added to the system (10 mg (~0.03 wt.% of the sol) or 50 mg (~0.15 wt.% of the sol)).

Samples containing 20% of APTMS were also tested as adsorbents; however, for most of the pollutants, there were no significant improvements in the removal efficiencies. Only in the case of amoxicillin was the presence of higher amounts of amine groups beneficial, so the results of the adsorption tests of these samples will only be presented in this case.

### 2.3. Characterization

Bulk density ($\rho_{b}$) was calculated by weighing and measuring portions of the sample. The volume of samples that can be cut into regular shapes was obtained by measuring their dimensions along three axes; the volume of irregular pieces was assessed by liquid displacement.

Helium pycnometry (Accupyc 1330, Micromeritics) was used to determine the skeletal density ($\rho_{s}$) of the aerogels. As per Equation (1), the bulk and skeletal densities were used to determine the porosity of the samples:

(1) Porosity%=1-ρbρs×100.

The Brunauer–Emmett–Teller (BET) specific surface area was obtained through nitrogen adsorption at 77 K (Gemini V2.00; Micromeritics Instrument Corp., Norcross, GA, USA), applying the BET theory in the relative pressure interval between 0.05 and 0.25 of the adsorption isotherms. Pore volume ($V_{P}$) and average pore size were calculated using Equations (2) and (3):

(2) $$V_{P}\left({cm}^{3}\cdot g^{-1}\right)=\frac{1}{\rho_{b}}-\frac{1}{\rho_{s}},$$

(3) $$Average\;pore\;diameter\;(nm)=\frac{4V_{P}}{S_{BET}},$$

The microstructures of the prepared samples were analyzed through scanning electron microscopy (SEM) using a Hitachi FEG-SEM SU70, operated at 15 kV, after being coated with a conductive carbon thin layer using a carbon rod coater (Emitech K950X).

Solid-State Nuclear Magnetic Resonance (SSNMR) analysis was performed to assess if any modification in the surface chemistry of the synthesized materials occurs after the adsorption of both drugs, amoxicillin, and naproxen. The SSNMR spectra were collected as described in Lamy-Mendes et al. [51].

### 2.4. Adsorption Experiments

After milling and sieving the prepared materials, adsorbents with particle sizes ranging from 75 to 250 µm were chosen for the adsorption tests. High purity water was utilized to make the adsorbate solutions. The adsorption tests were carried out by placing the adsorbent in a test flask and shaking it at 16 rpm and 20 °C with a Heidolph–REAX 20 shaker.

In the test flask, the adsorbent concentration was 2 g⋅L^−1^. The initial concentrations of the solutions varied between 10–500 mg⋅L^−1^ for benzene and its derivatives, and between 10–50 mg⋅L^−1^ for the drugs, for the equilibrium tests. The concentrations used were selected to be representative of the concentration range of organic compounds in industrial effluents [52,53,54]. To ensure that the equilibrium conditions were met, all solutions were shaken for 24 h. The kinetic experiments were carried out at various time intervals ranging from 2 to 4320 min. The solutions for benzene and its derivatives had an initial concentration of 100 mg⋅L^−1^, whereas the drugs had an initial concentration of 25 mg⋅L^−1^. Following the completion of each test, the solutions were filtered, and their concentration was determined by Ultraviolet–visible (UV-Vis) spectrophotometer (T70; PG Instruments, Lutterworth, UK) using specific wavelengths: 255, 262, 265, 270, nm for benzene, toluene, xylene, and phenol, respectively, and 273 and 272 nm, for amoxicillin and naproxen, respectively.

The equilibrium adsorption capacity, $q_{e}$ (mg⋅g^−1^), is defined by Equation (4), where $C_{0}$ (mg⋅L^−1^) and $C_{e}$ (mg⋅L^−1^) are the initial and equilibrium adsorbate concentrations, respectively, m (g) is the mass of the adsorbent and V (L) the volume of the solution:

(4) $$q_{e}=\frac{V(C_{0}-C_{e})}{m}.$$

The removal efficiency (*RE*) of the pollutants was calculated by Equation (5):

(5) $$RE\left(\%\right)=\frac{\left(C_{0}-C_{e}\right)}{C_{0}}\times 100.$$

To understand the interaction at equilibrium between the adsorbent and adsorbate, three models were studied in this work, the Langmuir, Freundlich and Brunauer–Emmett–Teller (BET) isotherm models. Non-linear fitting algorithms were used to fit the models to the data.

The Langmuir isotherm is described by Equation (6), where $q_{max}$ is the monolayer adsorption capacity of the adsorbent (mg⋅g^−1^) and $K_{L}$ is the Langmuir equilibrium constant (L⋅mg^−1^):

(6) $$q_{e}=\frac{q_{max}K_{L}C_{e}}{1+K_{L}C_{e}}.$$

The Langmuir adsorption isotherm model considers that the thickness of the adsorbed layer is one molecule, i.e., a monolayer adsorption, and that the adsorption process on the surface occurs at specific sites. The following premises are also assumed for this model: no lateral interaction occurs between the adsorbed molecules; all sites should have equal affinity regarding the adsorbate and that the surface is homogeneous [55]. The dimensionless constant known as separation factor ($R_{L}$), also referred as the equilibrium parameter, can be used to describe the features of the Langmuir isotherm. $R_{L}$ can be defined by Equation (7):

(7) $$R_{L}=\frac{1}{1+K_{L}C_{0}},$$

where $C_{0}$ is the initial concentration of the adsorbate in mg⋅L^−1^. The value of *R*_L_ indicates the shape of the isotherms to be either irreversible ($R_{L}$ = 0), favorable (0 < $R_{L}$ < 1), linear ($R_{L}$ = 1) or unfavorable ($R_{L}$ > 1) [55,56].

The Freundlich isotherm is described by Equation (8). The parameters *K*_F_ and *n*_F_ refer to the Freundlich constant ((mg⋅g^−1^) (L⋅mg^−1^)^1/*n*^_F_) and to the heterogeneity factor, respectively:

(8) $$q_{e}=K_{F}C_{e}^{1/n_{F}},$$

The Freundlich model is used to describe the reversible and non-ideal adsorption process, and, contrary to the Langmuir model, multilayer adsorption is possible. This model is also able to provide information about the adsorption mechanism; when the heterogeneity factor is higher than 1, cooperative adsorption occurs (multilayer), while if the 1/*n*_F_ is lower than 1, the process is favorable and the mechanism of sorption is mainly chemisorption [55,56].

The BET extinction model related to liquid–solid interface, which is characterized by the formation of an initial monolayer followed by a multilayer physical-based sorption [5,57], is exhibited in Equation (9), where $C_{BET}$, $C_{S}$, $q_{S}$ and $q_{e}$ are the BET adsorption isotherm relating to the energy of surface interaction (L⋅mg^−1^), adsorbate monolayer saturation concentration (mg⋅L^−1^), theoretical isotherm saturation capacity (mg⋅g^−1^) and equilibrium adsorption capacity (mg⋅g^−1^), respectively [58].

(9) qe=qSCBETCeCS-Ce1+CBET-1CeCS,

The BET model is considered a particular form of the Langmuir model, and the same suppositions are here applied, with the addition of the assumption that the same adsorption energy is found in the second, third, and higher layers. However, the first layer has a different energy than the other layers [55].

The adsorption rate was determined by fitting two kinetic models to the data, the pseudo-first and pseudo-second order models described by Equations (10) and (11) (after integration with appropriate boundary conditions) [59]:

(10) $$q_{t}=q_{e}\left(1-e^{{-tk}_{1}}\right),$$

(11) $$q_{t}=\frac{q_{e}^{2}k_{2}t}{q_{e}k_{2}t+1},$$

where k_1_ (1⋅min^−1^) and $k_{2}$ (g⋅(mg⋅min)^−1^) are the pseudo-first and pseudo-second order rate constants, respectively.

The Akaike’s information criteria (AIC), Equation (12), was the chosen methodology for the evaluation of the best model [60]:

(12) $$AIC=n\;log\left(\frac{s^{2}}{n}\right)+2K+\frac{2K(K+1)}{n-K-1},$$

where $s^{2}$ is the residual sum of squares; n is the number of experimental data points and K is the number of model parameters. The last term in AIC equation is added when the sample size is small (*n*/K < 40) to prevent an overfit [61].

Considering that individual AIC values are not interpretable the following re-scaling allows the comparison between models:

(13) $${∆}_{i}={AIC}_{i}-{AIC}_{min},$$

where ${AIC}_{min}$ is the minimum of the different AIC values. The ${∆}_{i}$ allows a meaningful interpretation: models with ${∆}_{i}$ ≤ 2 have a substantial support, those with 3 ≤ ${∆}_{i}$ ≤ 7 have considerably less support and the ones with ${∆}_{i}$ ≥ 10 have essentially no support [61,62].

## 3. Results and Discussion

### 3.1. Silica Aerogels Selection Based on Preliminary Adsorption Tests

The first step for the adsorbents’ screening was to determine which quantity of surfactant used during the synthesis procedures would lead to better removal rates for each pollutant. At the same time, the influence of amine groups in the silica matrix, with the addition of 10 mol percentage of APTMS, on the adsorption process was also tested. For these studies, only CTAB was used as a surfactant in order to reduce the number of experiments. These preliminary results are presented in Table 1.

For most of the adsorbate–adsorbent systems, the lower amount of CTAB (0.83 wt.%) presented a better adsorption performance, so, based on these data, from now on, only samples with this quantity of surfactant will be characterized and used for the remaining adsorption tests.

Regarding the silica systems, three organic compounds (benzene, toluene, and xylene) were better removed by the material with only MTMS in the matrix, probably due to their non-polar nature and the matrix’s super-hydrophobicity (contact angles higher than 150° [50]), which allows a hydrophobic interaction between the methyl groups derived from MTMS and these molecules.

The presence of amine improves the removal efficiency of phenol and amoxicillin, which can be explained by the interaction of the amine group in the matrix with the hydroxyl and carboxyl groups, in the case of AMX and NPX, via hydrogen bonding. For naproxen, both matrices presented similar results, so for this drug, the remaining tests will be performed with both silica systems, while for the other pollutants, only the best system will be used as an adsorbent.

### 3.2. Properties of the Adsorbents

The physical and microstructural properties of these materials are reported in Table 2. A more in-depth characterization of the carbon nanostructures–silica aerogel composites was performed in previous works from the authors [50,51].

The addition of 10 mol% Si from a silica precursor containing the aminopropyl groups into MTMS-silica materials leads to a small increase in the bulk density, as observed in Table 2. This minor deviation can be explained by the presence of aminopropyl groups, which, due to their basic character, can act as a basic catalyst during the synthesis, leading to a higher degree of condensation in the silica matrix. Additionally, amine-containing matrices cause greater capillary forces during drying due to a stronger retention of solvents, leading to greater shrinkage (higher density). However, all the carbon-nanotubes silica aerogel composite materials still have bulk densities values lower than 80 kg⋅m^−3^. While the addition of GO does not have a significant influence on this property, the presence of carbon nanotubes alters the bulk density, with small amounts of CNTs (10 mg) leading to a decrease in these values (Table 2), which can be justified by the fact that the CNTs reinforce the matrix, so the material tends to shrink less during the drying step. 

All the samples show porosities superior to 93% and pore volumes up to 15.6 cm^3^⋅g^−1^, with these results being similar to the ones obtained for their equivalents with 4.0 wt.% of surfactants [51]. The specific surface areas for the materials synthesized with only MTMS are the highest among the developed samples, and the obtained values are consistent with the literature, as organically modified silica (ORMOSIL) aerogels typically have values between 250 and 800 m^2^⋅g^−1^ [63].

For all the samples with APTMS, the addition of amine groups caused a significant decrease in the specific surface areas. The highest value among the 90M10A materials was obtained for the sample synthesized with CNT-HNO_3_, and one possible explanation for this variation can be the fact that these carbon nanotubes are not being completely surrounded by the silica matrix, contrary to the activity observed for CNT-TMOS, and their surface area is probably also contributing to the overall value. The lowest values were obtained for the GO samples.

**Figure 1:**
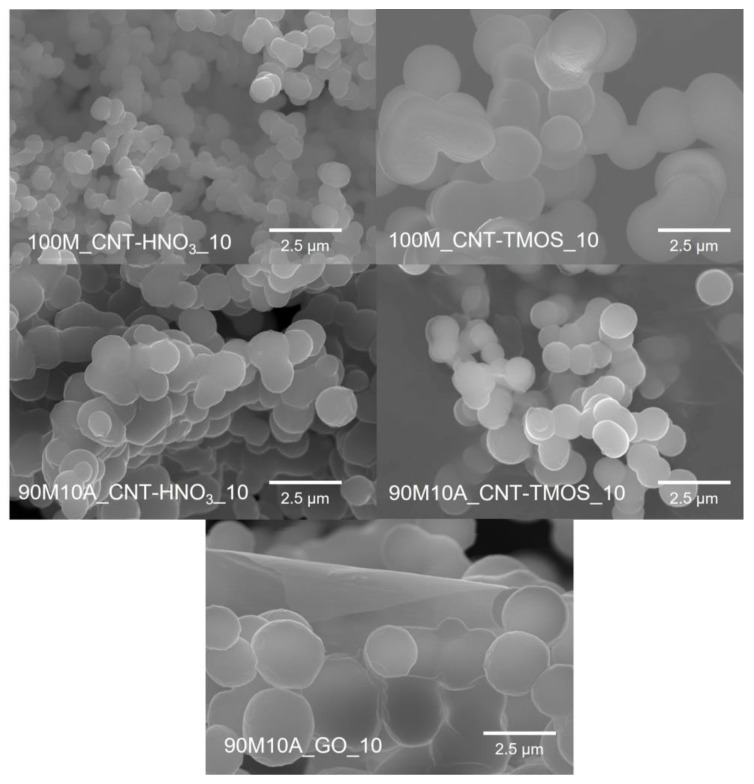
SEM images of the samples with 10 mg of CNTs-HNO_3_ or CNT-TMOS and with 10 mg of GO obtained with 0.83 wt.% of surfactant. (Magnification—×10.0 k).

Regarding the average pore sizes, the samples with lower surface areas presented the highest values as expected, with the samples with 10% of amine having pores in the micrometers order, except for 90M10A_CNT-HNO_3__10. However, this particular result can not be very accurate, as the average pore size is calculated from other properties (Equation (3)), including the specific surface area. In the case of the 90M10A_CNT-HNO_3__10 sample, a higher value of specific surface area was obtained, nonetheless, as already explained, this value probably has a contribution from the CNTs’ surface area, but the carbon nanotubes do not contribute to the formation of new pores or reduction in the existing ones, which leads to an underestimation of the pore sizes. Higher values of pore size are, in fact, supported by the SEM images (Figure 1), as this sample presents pore sizes in the same order than the other materials.

In the SEM images, Figure 1, it is possible to observe a significant amount of macropores in all the samples, which agree with the high values of pore sizes. For all composite materials, the characteristic structure of aerogels (pearl necklace) is observed. In the presence of carbon nanotubes, the samples synthesized with CNT-TMOS appear to have bigger secondary units than the composites obtained with CNT-HNO_3_. These differences probably occur because the silica matrix is able to grow around the silanized CNTs, while this is not verified for the samples with CNT-HNO_3_. Comparing the composites with CNT-HNO_3_, the one with only MTMS in the matrix seems to have smaller secondary particles than the 90M10A samples and appears to have a more uniform pore distribution. The larger secondary units were obtained for the GO composite, with them having almost double the size of the samples synthesized with CNTs. This sample also has the largest pores among the composite materials, which agrees with the results of surface area and average pore size presented in Table 2.

### 3.3. Study of Adsorption of Pollutants on the Selected Aerogels

#### 3.3.1. Benzene

**Figure 2:**
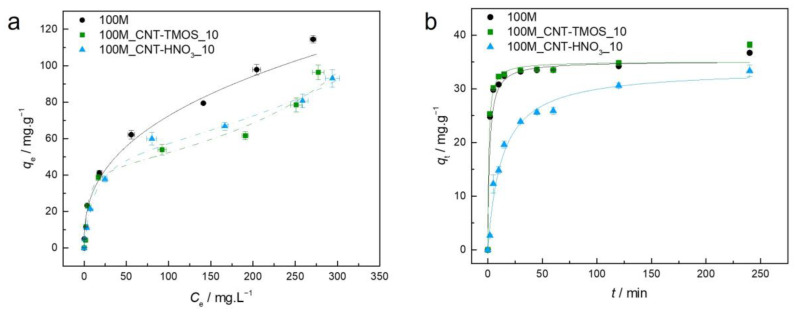
(**a**)—Experimental equilibrium data and the best fitted isotherm model for adsorption of benzene into the studied aerogels (solid line—Freundlich; dashed line—BET). (**b**)—Representative plots of the fit of nonlinearized form of pseudo-second order equation (solid line) to the data of experimental adsorption capacity as function of time, for the studied adsorbents towards benzene (*C*_0_ = 100 ppm, 15 rpm, 20 °C).

The parameters of the Langmuir, Freundlich, and BET isotherm models, as well as the pseudo-first and pseudo-second order kinetic models, are presented in Table S1 (Supplementary Materials) for the adsorbent–adsorbate pairs tested. The AIC was used as an estimator of the models’ relative quality. Experimental equilibrium and kinetic data and the best isotherm and kinetic models for each material are plotted in Figure 2.

For the sample 100M, the best fit was achieved by the Freundlich model, which describes adsorption for heterogeneous surfaces, and the heterogeneity factor obtained for the 100M sample indicates that the adsorption of this pollutant is favorable in this case, as also perceived by the shape of the isotherm.

When CNTs were added into the silica matrix, the BET model provided the most adequate results, indicating multilayer adsorption. However, in the case of sample 100M_CNT-HNO_3__10, the difference between the AIC values of the Langmuir and BET models was lower than two (Table S1), indicating that both models have statistical support. The Akaike weights were determined in order to select the best model among these, with the results providing the probability that the candidate model is the best among the set of models [64]. In this circumstance, the Akaike weight for the Langmuir model is 0.428 and for BET is 0.572, indicating that the BET isotherm model is 1.33 times more likely to be correct. However, it cannot be ruled out that the poor fitting of the Langmuir equation is due to the fact that *q*_e_ was not experimentally achieved, so higher concentrations of benzene would be required to confirm the adjustment. However, due to the toxicity of this compound, it was decided not to pursue the adsorption tests at concentrations higher than 500 ppm.

**Scheme 1:**
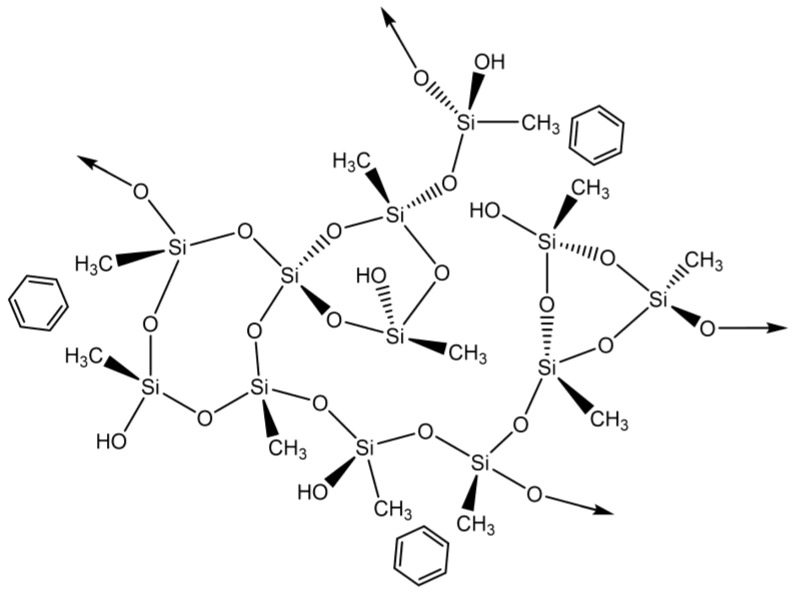
Proposed interactions between silica-based aerogels and benzene.

This pollutant’s adsorption mechanism is most likely based on a hydrophobic interaction (physical adsorption) between both the methyl groups derived from MTMS and benzene (Scheme 1).

The method used to prepare adsorbent materials can greatly affect their adsorption capabilities, as different synthesis processes result in distinct pore structures [65]. This influence is evident when comparing the benzene adsorption capacity of the MTMS material (100M) synthesized here with the one developed by Perdigoto et al. [65]. Despite the fact that the material here synthesized (100M) has a surface area value similar to that obtained by Perdigoto et al. [65], the adsorption capabilities reported herein were lower (114.4 mg⋅g^−1^), which can be attributed to the different pore structures, as the average pore size of 100M is 112.9 nm, whereas the silica-based material of Ref. [65] has a bimodal distribution of micropores and mesopores. To achieve higher benzene removal rates in MTMS-based materials, it appears that the presence of a significant proportion of both micropores and mesopores is critical. Nonetheless, the *q*_e_ observed here was greater than that obtained for other adsorbents such as HNO_3_-oxidized CNTs (105.7 mg⋅g^−1^) [66], cupric oxide nanoparticles (100.2 mg⋅g^−1^) [67] and PVA-PVP-Hap membrane (18.7 mg⋅g^−1^) [68] and β-cyclodextrin modified poly(butyl methacrylate) resin (16.3 mg⋅g^−1^) [69].

For the samples with both types of CNTs, BET was the best isotherm model. The BET model's underlying mechanism (multilayer adsorption) is supported by benzene’s ability to form aggregates via π–π stacking interactions [70]. For the carbon nanotubes–silica aerogel composites, the materials’ adsorption capacity shows a dependency on the pollutant’s initial concentration, with the *q*_e_ value increasing with the increase in the initial concentration of the adsorbate. These variations are in agreement with the occurrence of multilayer adsorption characterized by short-range interactions [5].

The addition of carbon nanotubes to the matrix caused a decrease in the removal efficiency of the MTMS-based matrix, which was not expected since modified CNTs have already been used for the removal of this pollutant and showed good performance [52,71]. One justification for the decrease in the removal capability, when CNT-TMOS were added to the composite, can be the fact that these nanotubes are surrounded by silica, since the matrix grows around these materials, as reported in previous works [50,51]. Thus, most of the CNTs are not really exposed, and, as consequence, they are not able to be in contact with the benzene solution. This supposition was reinforced as, for most of the pollutants here tested, no significant variation was observed when the silanized carbon nanotubes were added into the matrix, compared with the silica aerogels. The presence of CNT-HNO_3_ did not have a significant effect on the benzene removal; however, the presence of these CNTs leads to better results in the case of other pollutants, as will be shown in the following sections.

Regarding the kinetics, the 100M and 100M_CNT-TMOS_10 showed very similar profiles; however, the samples with the CNTs took a shorter time to achieve equilibrium (lower *k*_2_—Table S1). When CNT-HNO_3_ was used in the composite, a slower adsorption rate was obtained, and the equilibrium was reached in longer times than in the other samples. The experimental kinetic data of benzene adsorption in these silica-based aerogel composites were better explained by the pseudo-second order (PSO) model. The PSO kinetic model is used to describe both chemical and physical adsorptions [72,73]; as the isotherm models indicate that a physical adsorption occurs, it is a good indication that physisorption might dominate the adsorption process in this case, as suggested in Scheme 1. However, thermodynamic analyses are more appropriate for determining which process takes place (physisorption or chemisorption) [73], so additional information, such as activation energies, is necessary to conclude which one is the kinetic mechanism [74].

#### 3.3.2. Toluene

**Figure 3:**
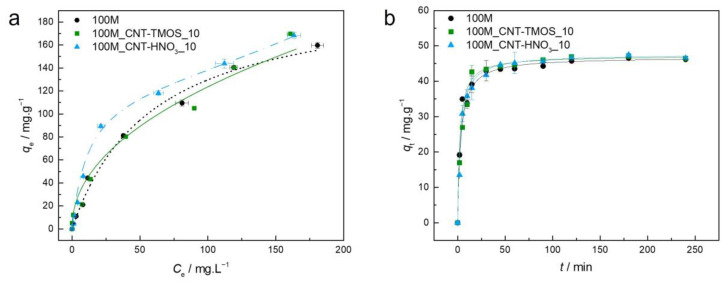
(**a**)—Experimental equilibrium data and the best fitted isotherm model for adsorption of toluene into the studied aerogels (dotted line—Langmuir; solid line—Freundlich; dashed line—BET). (**b**)—Representative plots of the fit of non-linearized form of pseudo-second order equation (solid line) to the data of experimental adsorption capacity as function of time, for the studied adsorbents towards toluene (*C*_0_ = 100 ppm, 15 rpm, 20 °C).

The parameters of the Langmuir, Freundlich, and BET isotherm models, as well as the kinetic models, are presented in Table S2, and the experimental equilibrium and kinetic data, and the best isotherm/kinetic models for each material regarding the adsorption of toluene, are plotted in Figure 3.

Regarding the adsorption of toluene, each material has a different isotherm model that provides a better fit; for the 100M, the model was Langmuir, suggesting that the adsorption occurs on a homogeneous surface and a monolayer of the sorbate is formed [56]. The model for the 100M_CNT-HNO_3__10 was BET, indicating that a multilayer adsorption takes place [55]. For the 100M_CNT-TMOS_10, the difference between the AIC values of the Freundlich and BET models was less than two, so once again the Akaike weights were used. The Freundlich model has an Akaike weight of 0.715, while BET has a value of 0.285, so the Freundlich is 2.51 times more likely to be the correct model in this case, with this model indicating that a favorable (1/*n*_F_ < 1) adsorption on heterogeneous surfaces happens [56].

**Scheme 2:**
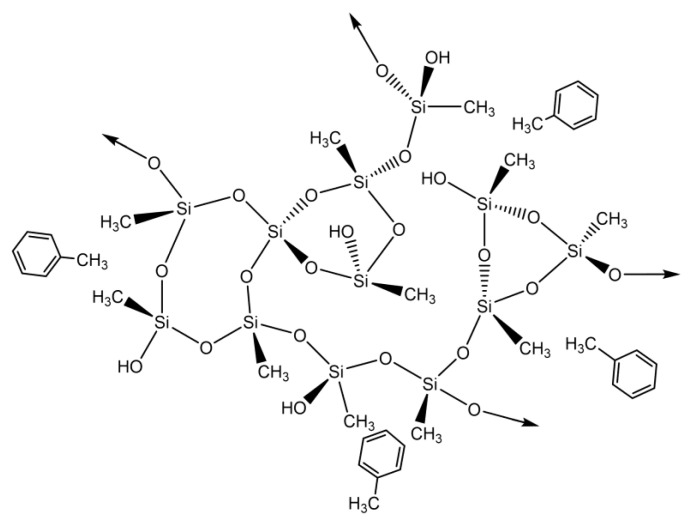
Proposed interactions between silica-based aerogels and toluene.

The results presented in Table S2 demonstrate that the addition of CNTs leads to higher experimental adsorption capacities than the MTMS-silica aerogel. The kinetic model that fitted all the samples better was again the pseudo-second order. As in the case of benzene, the removal of toluene from an aqueous solution by the silica materials is probably a physisorption process, as hydrophobic interactions are most likely to be occurring between the silica matrix and the pollutant, as represented in Scheme 2.

By observing the shapes of all the adsorption curves in Figure 3, it can be concluded that all materials have favorable adsorption isotherms. The same favorable behavior was observed in the work of Perdigoto et al. [65] that used MTMS aerogels and xerogel for toluene removal from aqueous solutions. Other hydrophobic adsorbents, including MTMS-derived aerogels and TMES/TMOS-derived aerogels obtained by Štandeker et al. [42] and silica aerogel granules (Cabot Nanogel^®^) used by Wang et al. [75], demonstrated unfavorable adsorption isotherms. It is important to mention that the majority of unfavorable isotherms are related to the adsorbents’ heterogeneous surfaces, which are directly correlated to the synthesis and drying of these materials [65].

For a better comparison, Wang and co-authors [75] estimated the adsorption capacity values for different materials when the *C*_e_ was 200 mg⋅L^−1^. The nanogel used in their work showed the lowest *q* (37 mg⋅g^−1^) among the analyzed materials, and the granulated activated carbon the highest (268 mg⋅g^−1^). The MTMS-based aerogels developed by Štandeker et al. [42] and by Perdigoto et al. [65] showed results of 87 mg⋅g^−1^ and 114 mg⋅g^−1^, respectively. The 100M_CNT-TMOS_10 has an adsorption capacity of 170 mg⋅g^−1^, which shows a significant improvement if compared with the other hydrophobic aerogels. This $q_{e}$ was also higher than the ones obtained with magnetic multi-walled carbon nanotube (MWCNTs) nanocomposite (63.3 mg⋅g^−1^) [76], magnetic zeolitic imidazole framework nanocomposites (118.4 mg⋅g^−1^) [77] and HNO_3_-oxidized CNTs (160.8 mg⋅g^−1^) [66].

As the best results were achieved by the composite materials, two more samples were developed with higher quantities of CNTs (50 mg), to determine if the amount of this carbon material would affect the removal efficiency of toluene from the aqueous solutions. The results obtained for four different initial concentrations are reported in Table 3.

For all concentrations, an increase in toluene’s removal from the solution was observed when the amount of silanized CNTs was raised to 50 mg, while for the CNT-HNO_3_ a slight decrease in efficiency was noticed. In these cases, the increase in carbon nanotubes in the systems does not have a significant effect on the adsorbent’s capacity, indicating that the variation in adsorption efficiency is probably more correlated with the impact that these carbon nanostructures have on the structure of the silica network, leading to a more branched network that can affect the average pore size and specific surface area, than with the presence of the carbon materials themselves.

#### 3.3.3. Xylene

**Figure 4:**
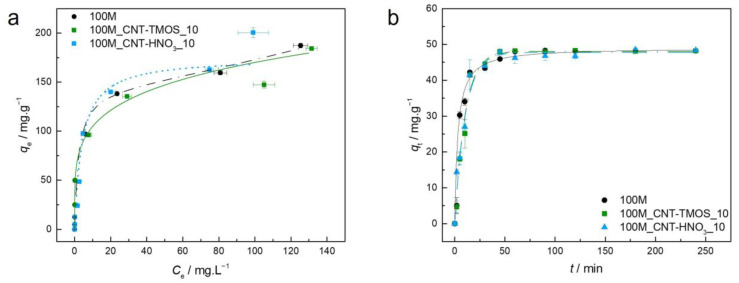
(**a**)—Experimental equilibrium data and the best fitted isotherm model for adsorption of xylene into the studied aerogels (dotted line—Langmuir; solid line—Freundlich; dashed line—BET). (**b**)—Representative plots of the fit of non-linearized form of pseudo-first order (dashed line) or pseudo-second order (solid line) equations to the data of experimental adsorption capacity as function of time, for the studied adsorbents towards xylene (*C*_0_ = 100 ppm, 15 rpm, 20 °C).

The parameters of isotherm and kinetic models are presented in Table S3, and the experimental equilibrium and kinetic data and the best isotherm/kinetic models for each material regarding the adsorption of xylene are plotted in Figure 4. Once again, for each material, the AIC indicates a different model as the best fitting; for the 100M it was the BET model, suggesting a multilayer adsorption, and for the 100M_CNT-TMOS_10 it was the Freundlich model, implying a favorable adsorption into a heterogeneous surface [55,56]. For the 100M_CNT-HNO_3__10, the Langmuir model showed the lower AIC value, however the Δ*_i_* is lower than two when compared with both other models, so the Akaike weight was assessed. As the Langmuir model has the lower AIC values, the Freundlich and BET models were compared with it. For Langmuir and Freundlich, the Akaike weights were 0.631 and 0.369, respectively, so Langmuir is 1.7 more likely to be correct. When comparing Langmuir with BET, the results were 0.549 and 0.451, respectively, and once again, Langmuir showed the best probability of being the correct model. As Langmuir provided the best fit to the data, that indicates that the adsorption of xylene by 100M_CNT-HNO_3__10 is favorable (0 < R*_L_* < 1) and a monolayer is formed.

Regarding the kinetics, the pseudo-second order model presented the best results for the silica aerogel, which was confirmed by the Akaike weights, with the PSO being 2.26 times more likely to be the most appropriate model, while both materials containing CNTs showed a best fit with the pseudo-first order (PFO), which indicates that the external/internal diffusion is the rate-limiting step [56]. The differences in the models that best described the kinetic data can be attributed to the different porous structure of the materials, as previously described, as the addition of carbon nanotubes affects the silica aerogel network, leading to distinct adsorption mechanisms [78]. The influence of structural differences was more evident in the kinetic mechanism of xylene, as PSO was the most appropriate model for benzene and toluene, and this can probably be explained by the differences in the kinetic energy, dipole moment, and spatial structure of this pollutant when compared to the other two.

**Scheme 3:**
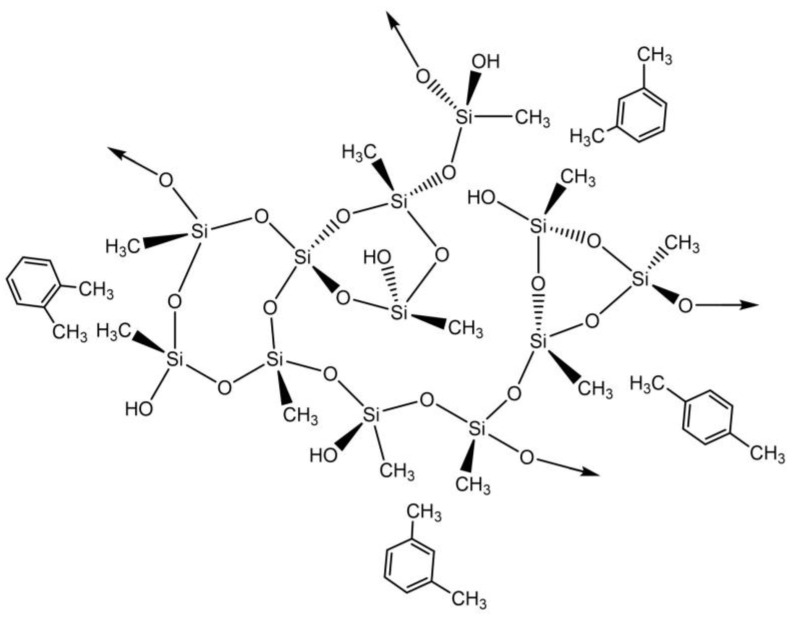
Proposed interactions between silica-based aerogels and xylene.

As suggested for benzene and toluene, the interaction between the xylene and the adsorbents is probably occurring by hydrophobic interactions, as shown in Scheme 3, which is in agreement with the results of the kinetic models, especially for the CNTs–silica aerogel composites, as the PFO model is used for the physical adsorption [72].

The highest $q_{e}$ experimental (200.5 mg⋅g^−1^) was obtained for the 100M_CNT-HNO_3__10 indicating an improvement with the addition of the carbon nanotubes, but all materials demonstrated good xylene removal from aqueous solutions. All these materials have removal rates of more than 90% for concentrations up to 300 mg⋅L^−1^, with sample 100M_CNT-HNO_3__10 having removal rates superior to 80% until concentrations of 500 mg⋅L^−1^. 

The $q_{e}$ here obtained was higher than the ones obtained for different materials, such magnetic ZSM zeolite (*p*-xylene—108.11 mg⋅g^−1^) [79], HNO_3_-oxidized CNTs (108.9 mg⋅g^−1^) [66], single wall carbon nanotubes–magnetic nanoparticle [80] (50 mg⋅g^−1^) and zeolite from fly ash [81] (*p*-xylene—0.129 mg⋅g^−1^ and o-xylene—0.147 mg⋅g^−1^). Yu et al. [76] developed a magnetic multi-walled carbon nanotube (MWCNTs) nano-composite for xylene removal, reaching adsorption capacities of 227.2 mg⋅g^−1^ for *m*-xylene, 138.1 mg⋅g^−1^ for *o*-xylene and 105 mg⋅g^−1^ for *p*-xylene. Even though one of the values is slightly higher than the ones obtained for 100M_CNT-HNO_3__10, it is important to mention that Yu et al. executed the tests in individual isomer solutions while our adsorption tests were performed in a solution containing a mixture of xylene isomers, which can influence the final adsorption capacities.

As better results were achieved in the presence of CNTs, the materials containing 50 mg of CNTs in the MTMS–silica matrix were also tested for the removal of xylene. The results obtained for these pollutants are presented in Table 4.

As observed in Table 4, for concentrations up to 200 mg⋅L^−1^, all these materials have similar removal efficiencies, being able to remove more than 95% of the pollutant from the aqueous solutions. Only for the highest tested concentration was a difference noticed, with the 100M_CNT-HNO_3__10 sample having the best performance. As observed in toluene’s case, the increase in the amount of CNTs does not have a significant impact on the removal of xylene, indicating that 10 mg of CNTs is an appropriate amount for the MTMS-based silica matrix.

The interaction between the hydrophobic surface of the MTMS aerogels and the adsorbate molecules is different for each organic compound. For the materials developed here, toluene and xylene are adsorbed to a greater extent than benzene, as both have higher maximum adsorption capacities for all adsorbent/adsorbate pairs. These differences can be correlated with the different dipole moments and steric hindrance, which allow better interaction between the matrix and the benzene derivatives than the silica materials with benzene itself.

#### 3.3.4. Phenol

**Scheme 4:**
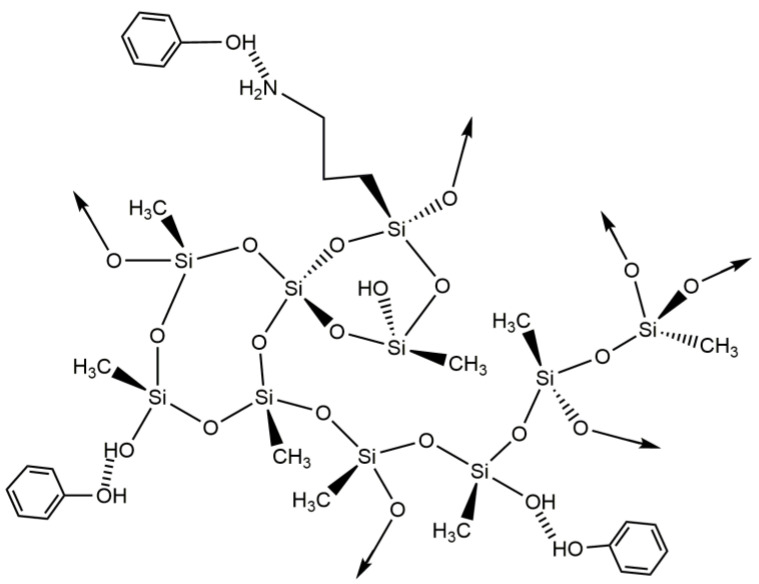
Proposed interactions between silica-based aerogels and phenol.

Benzene and its derivatives have so far been better adsorbed by materials in which the amine groups are absent. However, the best phenol removal efficiency was observed with the 90M10A adsorbent when compared to the 100M, for an initial concentration of 200 mg⋅L^−1^, as seen in Table 1. This could be explained by the hydrogen bonding interaction between the amine groups of the MTMS/APTMS-based aerogel and the hydroxyl group of the phenol molecules, as shown in Scheme 4. When compared to the pure MTMS-based material, it is noticeable that the $q_{e}$ is enhanced by the incorporation of amine groups.

**Figure 5:**
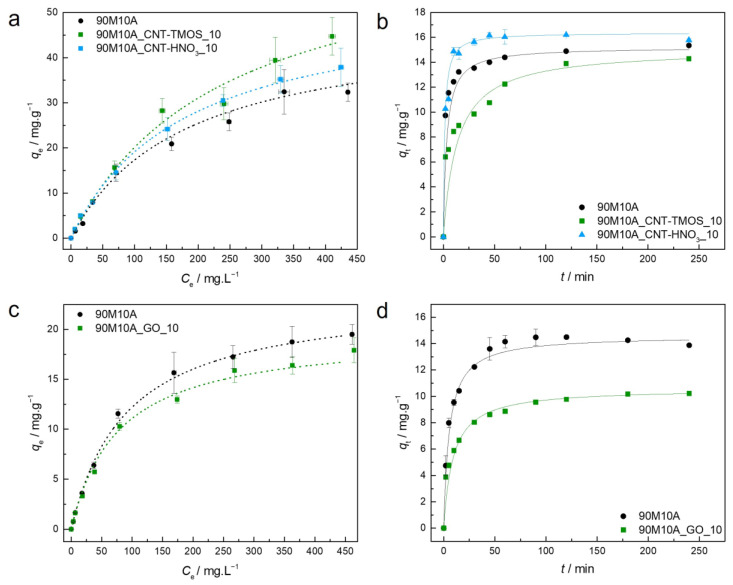
(**a**,**c**)—Experimental equilibrium data and the best fitted isotherm model for adsorption of phenol into the studied aerogels (dotted line—Langmuir). (**b**,**d**)—Representative plots of the fit of non-linearized form of pseudo-second order equation (solid line) to the data of experimental adsorption capacity as function of time, for the studied adsorbents towards phenol (*C*_0_ = 100 ppm, 15 rpm, 20 °C).

As the presence of amine groups provided better removal efficiency for phenol, it was possible to also test the adsorption capacities for the composite materials made with graphene oxide. The parameters of the isotherm and kinetic models are presented for the composites with carbon nanotubes in Table S4 and for the materials developed with graphene oxide in Table S5. The experimental equilibrium and kinetic data, along with the best isotherm/kinetic models for each material regarding the adsorption of phenol, are plotted in Figure 5.

For all the materials tested for phenol removal, the BET model was not able to provide an adjustment to the data, with the calculated parameter values having no physical meaning, so these data were not presented. For phenol adsorption, the Langmuir model explains the interactions of all the materials more effectively, including the batch of GO-silica aerogel composites. The data for the 90M10A_CNT-HNO_3__10 were also confirmed by Akaike weights, with the Langmuir model being 2.5 times more probable to be correct if compared with Freundlich (Table S4). This isotherm model indicates that monolayer surface adsorption occurs at specific sites. According to the isotherms’ shapes, and the RL parameter (Tables S4 and S5), the adsorption of this pollutant by the silica-based adsorbents is favorable, which agrees with the conclusions of Matias et al. [82].

Comparing both systems developed here, when PEG was used as surfactant (GO-silica aerogel composites), a lower maximum $q_{e}$ was obtained, around half of the results for the samples with CTAB. One possible explanation is that these PEG samples have lower surface areas than the ones synthesized with CTAB (Table 2), which can have a significant impact on the number of available sites for the adsorption of phenol.

The CNTs–silica aerogel composites exhibit higher $q_{e}$ values (44.7 mg⋅g^−1^ and 37.9 mg⋅g^−1^ for the materials with CNT-TMOS and CNT-HNO_3_, respectively) than different adsorbents, such as granular-activated carbon (1.48 mg⋅g^−1^) [83], calcinated clay (2.9 mg⋅g^−1^) [84], MTMS xerogels (4.9 mg⋅g^−1^) [65], porous hydroxyapatite (8.2 mg⋅g^−1^) [85], reduced graphene oxide (16.1 mg⋅g^−1^) [86], graphene oxide (19.2 mg⋅g^−1^) [86], MTMS aerogel (21.1 mg⋅g^−1^) [65] and iron oxide (21.9 mg⋅g^−1^) [87]. The GO composites were also able to outperform most of the previously mentioned materials in terms of $q_{e}$.

For all the materials removing phenol, the pseudo-second order model provided the best fitting to the data (Figure 5b,d). For most of the composites here developed, when adsorbing organic compounds from aqueous solutions, the pseudo-second order fits well with the data. The kinetic data, combined with the isotherm results, as Langmuir provides the best adjustment to the data, are compelling in demonstrating that the chemisorption is the dominant mechanism occurring for these adsorptions’ systems, as demonstrated by Matias et al. [82], who used MTMS-based systems modified with Glymo and β-cyclodextrin for phenol removal. However, considering the silica matrix here used, hydrogen bonds are the most likely interaction between the pollutant and the adsorbent, as indicated in Scheme 4. This discrepancy between theory and experimental data is most likely because no clear distinction between chemisorption and physisorption can be made without additional thermodynamic tests, particularly in cases involving strong hydrogen bonds.

As the best results were obtained for the samples with carbon nanotubes, once again composites with 50 mg were tested to verify the influence of the quantities of these CNTs in the removal of the pollutant, in this case phenol, with these results being presented in Table 5.

In the phenol case, better results were obtained for lower initial concentrations with 10 mg of CNTs, as verified for the other organic compounds; however, when the concentration is 400 mg⋅L^−1^, the increase to 50 mg of CNTs in the silica matrix is beneficial for the removal of this pollutant from aqueous solutions, the difference being more expressive with CNT-HNO_3_ (more exposed). One possible explanation for this is that, if the pollutant has a better affinity with the silica matrix than with the carbon nanostructure, only with higher pollutant concentrations can the amount of CNTs start to make a difference, as the active sites on the silica will be already saturated. 

For the same initial concentration (200 mg⋅L^−1^), the removal efficiencies of these aerogel composites were higher than those obtained by Matias et al. [82], who used MTMS-based systems modified with Glymo and β-cyclodextrin for phenol adsorption (lower than 20%), and from our previous work [49], which used the same silica matrix but with a higher amount of surfactant during the synthesis procedure (19.1%). 

#### 3.3.5. Amoxicillin

As already mentioned, drugs are relevant pollutants in wastewater, so their removal is important to assure the safety of water bodies. The first tests for drug removal from aqueous solutions were performed with amoxicillin, using adsorbents with 90% MTMS and 10% APTMS. However, for this particular pollutant, better results were obtained when adding higher amounts of APTMS, so in this case, composites using 90M10A and 80M20A matrices were used as adsorbents.

**Figure 6:**
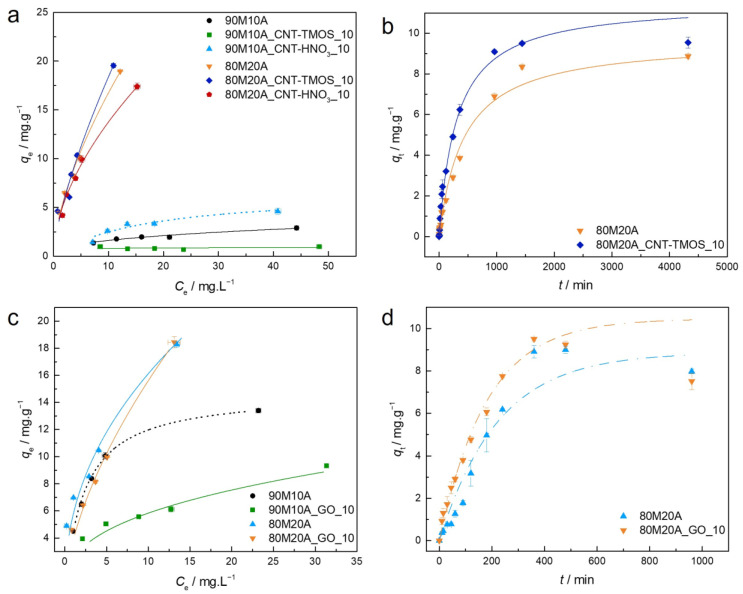
(**a**,**c**)—Experimental equilibrium data and the best fitted isotherm model for adsorption of amoxicillin into the studied aerogels (dotted line—Langmuir; solid line—Freundlich). (**b**,**d**)—Representative plots of the fit of non-linearized form of pseudo-first order (dashed line) or pseudo-second order (solid line) equations to the data of experimental adsorption capacity as function of time, for the studied adsorbents towards amoxicillin (*C*_0_ = 25 ppm, 15 rpm, 20 °C).

The parameters of isotherm models are presented for the composites with carbon nanotubes in Tables S6 and S7, and the results for the materials developed with graphene oxide are shown in Table S8. The kinetic tests were performed only for the materials with better removal efficiency, and the parameters for the kinetic models are displayed in Table S9. For all the adsorbents, the BET model was not able to provide any adjustment to the data, providing values that have no physical meaning, so for AMX these data will not be presented. The experimental equilibrium and kinetic data, with the best isotherm models for each material, and the kinetic models for some of the adsorbents regarding the removal of AMX, are plotted in Figure 6.

For the carbon nanotubes–silica aerogel composites, the isotherms were better described by the Freundlich model, confirmed by the Akaike weights. As previously mentioned, the Freundlich model describes adsorption on heterogeneous surfaces, and the values obtained for the heterogeneity factor indicate a favorable adsorption [55]. The only exception was the sample 90M10A_CNT-HNO_3__10, for which the Langmuir model provided the best results, suggesting monolayer adsorption. For the GO–silica aerogel composites, the data for silica matrix with 10% of APTMS were better described by the Langmuir model, while all the other samples show better fittings with the Freundlich isotherm model. As already mentioned, for these composites, a matrix with 20% of APTMS provided superior removal of AMX from the tested solution, but the presence of graphene oxide in the tested amount (10 mg) did not contribute to a better performance.

Regarding the kinetics, in the presence of CNTs, the PSO model provided the best fit to the data, while for the GO composites, the best fit was achieved by the PFO model. This divergence indicates that these materials can interact with the pollutant differently, as PSO is used to describe chemical as well as physical adsorptions [72,73], while PFO suggests that a physisorption mechanism takes place. If compared to the organic compounds previously tested, the AMX kinetic is much slower, only achieving equilibrium after 1000 min in the case of CNTs-composites, and after 400 min for the samples with GO.

**Figure 7:**
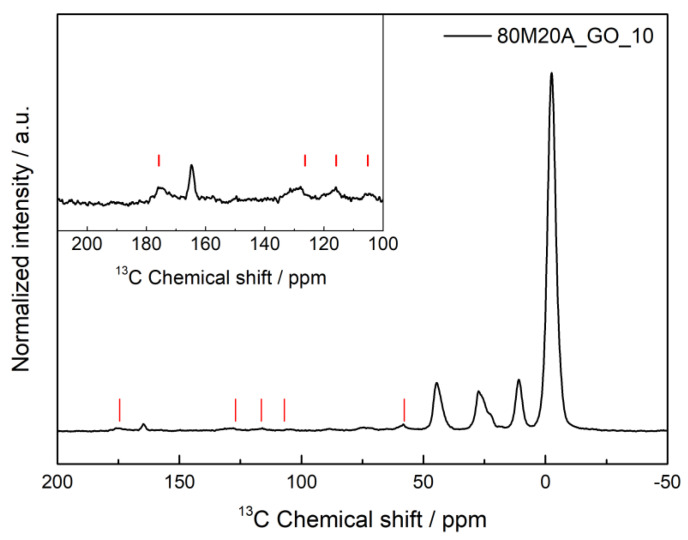
^1^H–^13^C heteronuclear solid-state NMR spectra of 80M20A_GO_10 after the adsorption process of amoxicillin. The red lines indicate the detected AMX peaks. The inset shows a vertical expansion of the spectral range where the AMX peaks are present.

To confirm if any chemical interactions between the composite materials and the amoxicillin molecule occurred during the adsorption process and provide a better indication as to whether physisorption or chemisorption is happening in the adsorption process, SSNMR was performed, and the result obtained for the 80M20A_GO_10 after the adsorption process of AMX is represented in Figure 7.

**Scheme 5:**
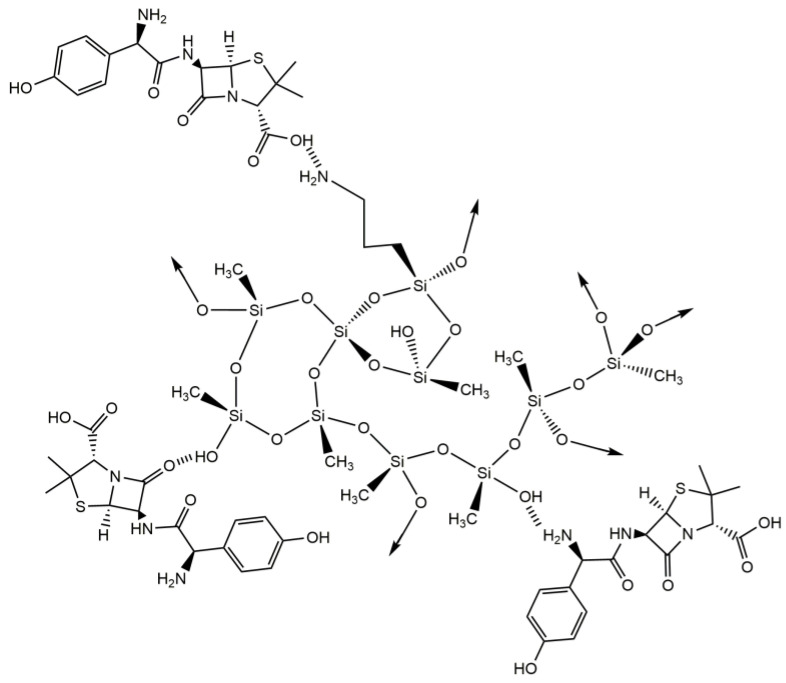
Proposed interactions between silica-based aerogels and amoxicillin.

In Figure 7, it is possible to observe the peaks arising from the silica matrix, with the carbons from the aminopropyl group appearing at 11, 27 and 45 ppm, and the main Si-CH_3_ signal at −3 ppm. Some characteristic peaks of amoxicillin were also detected in the spectra, around 57 ppm, 107 ppm, 116 ppm, 126–129 ppm and 174 ppm. No obvious sign of drug–GO interaction was verified, and the same pattern was observed for the AMX adsorption in composites with carbon nanotubes. This indicates that the NMR data could not detect the formation of a chemical bond between the AMX and the silica aerogel; however, the detection of AMX peaks proves that the adsorption of the drug was effective in the composites by physisorption. Therefore, it is possible to assume that hydrogen bonds, between the hydroxyl and amino groups of the silica aerogel and the three different groups of AMX (hydroxyl, carboxyl, and amino groups) are the most likely interaction between them, as indicated in Scheme 5.

In the work developed by Pouretedal and Sadegh [88], the maximum sorption was achieved when 20 mg⋅L^−1^ of initial concentration was used, with the activated carbon nanoparticles prepared from vine wood being used as adsorbent, and having a removal efficiency of around 72% of AMX from the solution. For the same concentration, both carbon nanostructure–silica aerogel composites here developed (80M20A_CNT-TMOS_10 and 80M20A_GO_10) were able to achieve higher removal rates (around 82%). De Franco et al. [89] also used activated carbon to remove AMX from aqueous solutions and achieved a *q*_max_ of 4.4 mg⋅g^−1^, a much lower result than the ones obtained for our materials (up to 19.5 mg⋅g^−1^ for the composites with CNTs and up to 18.4 mg⋅g^−1^ for the materials with GO). The adsorption capacities here achieved were also higher than the ones obtained for AMX removal with Zr-MOFs nanoparticles (2.3 mg⋅g^−1^) [16], MnO_2_@carbon microspheres (16.1 mg⋅g^−1^) [90] and modified montmorillonite (13.3 mg⋅g^−1^) [91].

However, our results were lower than the ones presented by Moussavi et al. [92], which applied NH_4_Cl-induced activated carbon for AMX removal, with this material being able to remove over 99% from an initial amoxicillin solution with a concentration of 50 mg⋅L^−1^. The better performance of their material can be attributed to its significantly larger surface area (1029 m^2^⋅g^−1^), which can have an important influence on the material’s adsorption capacity.

The next step was to verify if the amount of carbon nanostructure has any influence in the AMX removal. The quantities of CNT-TMOS and GO were increased in the samples 80M20A. The results are shown in Table 6.

In the case of carbon nanotubes, the increase to 50 mg improved the removal efficiency of AMX until a concentration of 25 mg⋅L^−1^. However, for the highest *C*_0_, the removal efficiency remained practically the same. For the GO composites, the variation did not cause a meaningful alteration. As observed for the organic compounds, better AMX removal efficiency was achieved when 10 mg of carbon nanostructure was added into the silica matrix.

#### 3.3.6. Naproxen

**Scheme 6:**
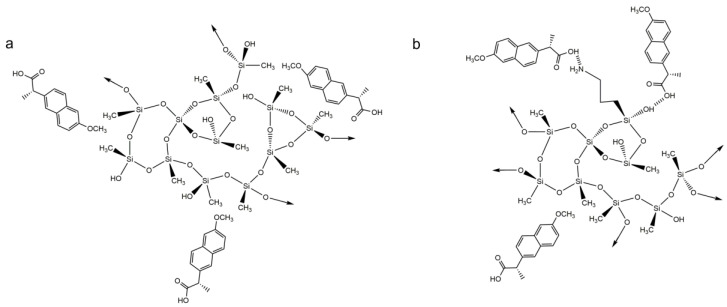
Proposed interactions between naproxen and (**a**) 100% MTMS silica aerogels and (**b**) 90% MTMS and 10% APTMS silica aerogels.

The last pollutant tested with the carbon nanostructure–silica aerogel composites as adsorbents was naproxen. As already mentioned, both silica matrices were able to successfully remove NPX from aqueous solutions, so it is possible that distinct parts of the naproxen molecule are interacting with the different matrices, with hydrophobic interactions taking place between their methyl groups in the MTMS–silica aerogel (Scheme 6a), while for the matrix containing APTMS, in addition to these, the drugs’ carboxyl groups can make hydrogen bonds with the silanol and amine groups of the aerogel (Scheme 6b).

**Figure 8:**
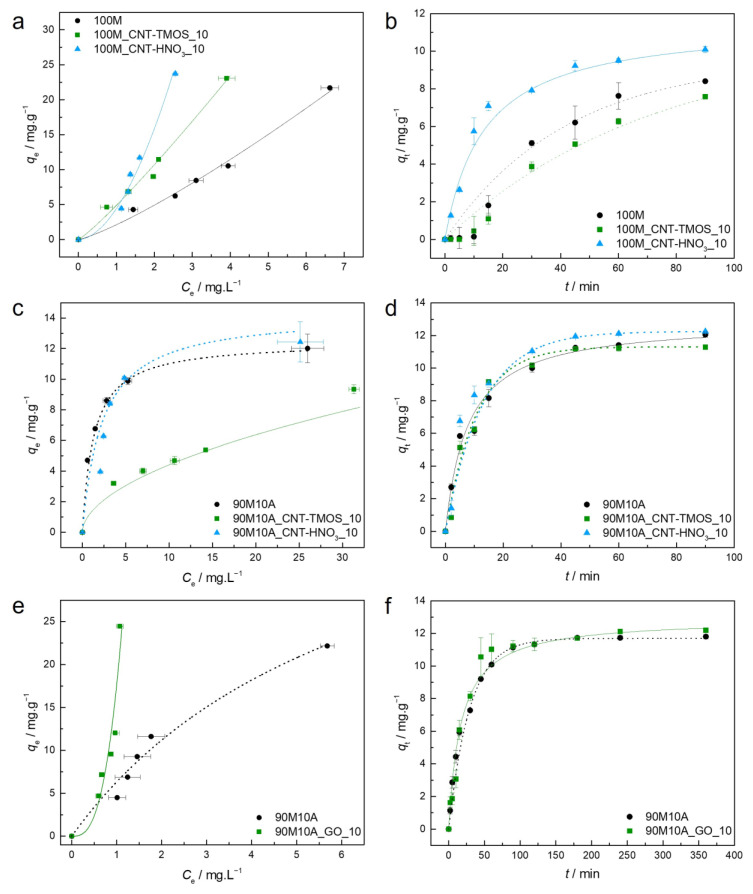
(**a**,**c**,**e**)—Experimental equilibrium data and the best fitted isotherm model for adsorption of naproxen into the studied aerogels (dotted line—Langmuir; solid line—Freundlich). (**b**,**d**,**f**)—Representative plots of the fit of non-linearized form of pseudo-first order (dashed line) or pseudo-second order (solid line) to the data of experimental adsorption capacity as function of time, for the studied adsorbents towards naproxen (*C*_0_ = 25 ppm, 15 rpm, 20 °C).

The parameters of isotherm and kinetic models are presented for the 100M with carbon nanotubes in Table S10, for the 90M10A with CNTs in Table S11 and for the 90M10A with GO in Table S12. For the samples with 100M and the 90M10A_GO_10, the Langmuir model presented fit parameters with no physical meaning, so they are not presented in the tables. For the samples 100M_CNT-HNO_3__10 and both samples with GO, the BET model was not able to adjust the data, so they are also not displayed in the tables. The experimental equilibrium and kinetic data, with the best isotherm models for each material and the kinetic models for the adsorbents regarding the removal of NPX, are plotted in Figure 8.

For the 100M systems, the Freundlich model provides the best fit to the data. The isotherms obtained from the adsorption process are unfavorable (Figure 8a), as shown by the values of 1/*n*_F_ (Table S10). For these samples, the addition of CNT leads to an increase in the maximum *q*_e_. While the CNT-HNO_3_ allowed the equilibrium to be reached in relatively shorter times, with the CNT-TMOS a decrease in the kinetic constant was observed. For the silica sample and the composite with CNT-TMOS, PFO provided a better adjustment to the data, while for the 100M_CNT-HNO_3__10, PSO showed better results. According to Vareda et al. [56], when the heterogeneity factor is higher than one, as the ones here obtained, a cooperative (multilayer) adsorption occurs, which is in agreement with the kinetic results, as both models can represent the physisorption mechanism. Therefore, for the materials developed with only MTMS as precursor, no chemical interaction takes place during the adsorption process, which agrees with the proposed adsorption mechanisms presented in Scheme 6a.

For the 90M10A batch of samples with CNTs, the AIC showed that for 90M10A and 90M10A_CNT-HNO_3__10, the Langmuir model has the best adjustment to the data, with the shape of the isotherms (Figure 8) and the RL values (Table S11) indicating a favorable adsorption. For the composite with CNT-TMOS, the Freundlich model has the best results, and also has a favorable adsorption profile (Table S11 and Figure 8). The highest values of *q*_e_ were once again achieved by the composite with CNT-HNO_3_. For the silica aerogel, pseudo-second order was the preferred kinetic model, while in the presence of CNTs, the best fit was obtained for the pseudo-first order, once again indicating that the adsorption is probably physical, and they all achieved equilibrium in similar times.

In the case of graphene oxide composites, the addition of GO leads to an improvement in the *q*_e_ and the data were adjusted by the Freundlich model, while the data of its silica counterpart were better fitted by the Langmuir model. The presence of GO changed the shape of the isotherm from favorable to unfavorable, as shown by the 1/*n*_F_ value (Table S12). The silica aerogel behavior is better described by the PFO model, while the PSO was more appropriate for the data of the GO–silica aerogel composite.

Comparing the maximum experimental *q*_e_, the 100M samples with CNTs and the 90M10A with GO have similar values and are higher than the ones for the 90M10A matrix with CNTs. Therefore, for the study of the influence of carbon nanostructure amount, these three matrices were used. The removal efficiencies for different concentrations of NPX are reported in Table 7.

The best results were achieved by the samples containing 50 mg of graphene oxide in the silica matrix, with removals superior to 96% up to an initial concentration of 50 mg⋅L^−1^. The removal efficiency of naproxen obtained for 90M10A_GO_50 is higher than those reported for magnetic-activated carbon (87.8%) [93], Fe_3_O_4_ nanoparticles on multi-walled carbon nanotubes (67.2%) [93] and functionalized nano-clay composite (92.2%) [94], all having an initial concentration of 10 mg⋅L^−1^. For *C*_0_ of 20 mg⋅L^−1^, the GO–silica aerogel composites (96.2%) still have better performance than other materials, such as olive waste cakes (~95%) [95] and amberlite XAD-7 (~60%) [96]. The composite here developed also presented a higher adsorption capacity (23.7 mg⋅g^−1^) than other adsorbents, such as 3D gelatin-based aerogels (8.5 mg⋅g^−1^) [97], granular-activated carbon (3.2 mg⋅g^−1^) [98] and molecularly imprinted polymer (4.5 mg⋅g^−1^) [99].

**Figure 9:**
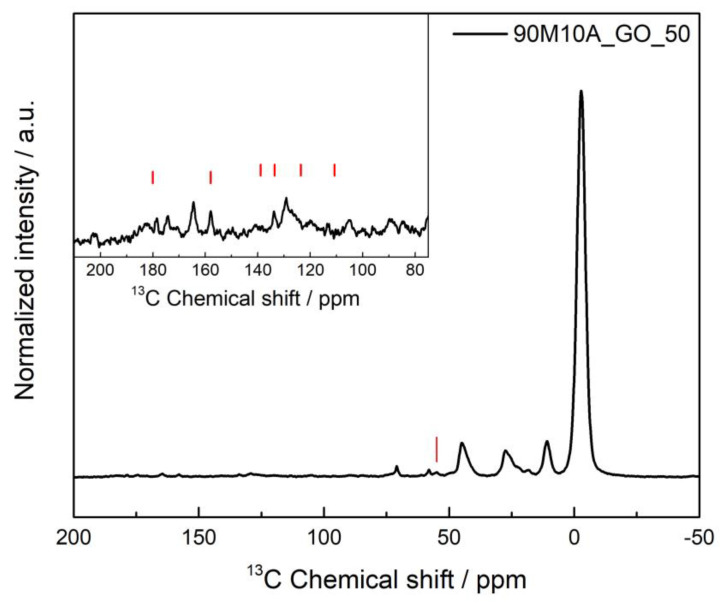
^1^H–^13^C heteronuclear solid-state NMR spectra of 90M10A_GO_50 after the adsorption process of naproxen. The red lines indicate the detected NPX peaks. The inset shows a vertical expansion of the spectral range where the NPX peaks are present.

As the highest removal efficiency was achieved for the 90M10A_GO_50 composite, SSNMR was performed for this sample after the adsorption of naproxen to verify if any interaction between adsorbent–adsorbate occurred during this experiment. The NMR spectrum is represented in Figure 9. 

By analyzing the spectrum, it was possible to observe the peaks from the silica matrix, as described previously, and, besides that, NPX is clearly detected, with its characteristic peaks around 55 ppm, 105 ppm, 119 ppm, 126–133 ppm, 134 ppm, 158 ppm and 180 ppm. The intensities qualitatively correspond to the concentration of the solution removed. It is possible that a specific interaction occurs during the adsorption process, as shown by the appearance of three peaks instead of only one around 180 ppm, but the low signal-to-noise in the spectra precludes a more detailed analysis of the possible interactions. The arising of these peaks can indicate an interaction between the carboxyl group of naproxen, for which the carbon appears at 180 ppm, with the graphene oxide. This theory, and the possibility of *π*–*π* interaction between graphene oxide and benzene ring of naproxen, can explain the higher sorption efficiency of this material.

## 4. Conclusions

The ability to modify the properties of silica aerogels allows these adsorbents to be tailored to better remove various pollutants from wastewater. In this work, we were able to verify that the presence of amine groups has a significant impact on the materials’ adsorption capacities. The presence of carbon nanostructures also led to an increase in the removal performance of the silica aerogels for most of the contaminants. However, without further investigation, it is not possible to affirm that this was due to adsorption on the carbon nanostructures or to the structural changes that they cause in the silica network, which may lead to the formation of new active sites.

The MTMS-based aerogels effectively removed non-polar organic compounds such as benzene, toluene, and xylene. In the presence of carbon nanotubes, removal efficiencies greater than 70% were reached for concentrations of toluene up to 400 mg⋅L^−1^, with similar results also being obtained for xylene. In the case of phenol, the presence of amine had a significant impact on removal efficiency. The addition of APTMS to both amoxicillin and naproxen was also critical for improved performance. Removal efficiencies higher than 70% were achieved for AMX for concentrations up to 50 mg⋅L^−1^, while in the case of NPX, the removals were always higher than 94% for the composites containing amino groups. 

Following this analysis, it is worth noting that the presence of amine groups and carbon nanostructures is a valuable tool for the development of new adsorbents by altering their properties to improve the adsorption of relevant pollutants. As a result, this work clearly demonstrates that these materials have a high potential for use as alternative industrial sorbents due to their higher and faster removal efficiency against various types of pollutants.

## Figures and Tables

**Table 1:** Silica precursor system, amounts of CTAB used during the synthesis and the removal efficiency of the materials for the studied pollutants.

		Removal Efficiency (%)			
Pollutant	*C*_0_ (mg⋅L^−1^)	MTMS		90MTMS/10APTMS	
		0.83 wt.%	4.0 wt.%	0.83 wt.%	4.0 wt.%
Benzene	200	72.1 ± 2.1	51.0 ± 1.4 [49]	50.0 ± 2.0	15.4 ± 1.6 [49]
Toluene	200	81.1 ± 0.7	68.9 ± 2.3	48.7 ± 2.4	51.7 ± 1.9
Xylene	200	96.9 ± 1.4	86.7 ± 0.1	66.9 ± 0.8	74.8 ± 0.7
Phenol	200	20.0 ± 2.4	8.4 ± 0.8 [49]	25.9 ± 1.2	19.1 ± 0.9 [49]
Amoxicillin	25	5.3 ± 2.0	5.0 ± 2.3	15.6 ± 1.3	19.4 ± 2.5
Naproxen	25	84.2 ± 0.7	69.0 ± 0.9	79.0 ± 1.8	93.8 ± 0.3

**Table 2:** Summary of physical and microstructural properties of the synthesized silica aerogels and carbon nanostructures–silica aerogel composites with 0.83 wt.% of surfactant. Adapted with permission from ref. [50]. Copyright (2019) Elsevier.

Samples	Bulk Density (kg⋅m^−3^)	Porosity (%)	Pore Volume (cm^3^⋅g^−1^)	Specific Surface Area (m^2^⋅g^−1^)	Average Pore Size (nm)
100M	75.6 ± 2.8	94.8 ± 0.1	12.5 ± 0.5	444.6 ± 4.3	112.9 ± 3.4
100M_CNT-HNO_3__10	73.1 ± 2.8	93.9 ± 0.3	12.8 ± 0.7	465.4 ± 5.7	110.4 ± 7.7
100M_CNT-TMOS_10	61.3 ± 5.3	95.8 ± 0.2	15.6 ± 1.4	463.4 ± 4.5	134.9 ± 10.1
90M10A	79.3 ± 11.1	94.6 ± 0.7	11.9 ± 1.8	11.3 ± 0.2	4221 ± 546
90M10A_ CNT-HNO_3__10	76.3 ± 2.5	95.2 ± 0.1	12.5 ± 0.4	293.7 ± 3.0	170.0 ± 7.3
90M10A_CNT-TMOS_10	76.2 ± 5.6	95.1 ± 0.3	12.5 ± 1.0	18.4 ± 0.3	2713 ± 180
90M10A	83.0 ± 2.8	93.9 ± 0.2	11.3 ± 0.4	5.02 ± 0.9	9021 ± 1929
90M10A_GO_10	79.2 ± 1.2	94.3 ± 0.1	11.9 ± 0.2	11.2 ± 0.2	4250 ± 140

**Table 3:** Removal efficiency of the carbon nanotubes–silica aerogel composites with two amounts of carbon nanostructure for different initial concentrations of toluene.

	Removal Efficiency (%)			
	*C*_0_ = 50 mg⋅L^−1^	*C*_0_ = 100 mg⋅L^−1^	*C*_0_ = 200 mg⋅L^−1^	*C*_0_ = 400 mg⋅L^−1^
100M_CNT-TMOS_10	85.2 ± 1.3	86.4 ± 1.4	80.2 ± 0.7	70.1 ± 0.3
100M_CNT-TMOS_50	88.5 ± 0.2	92.8 ± 0.3	85.6 ± 0.1	71.9 ± 0.5
100M_CNT-HNO_3__10	91.7 ± 0.4	91.8 ± 0.4	89.4 ± 1.3	71.9 ± 1.7
100M_CNT-HNO_3__50	91.9 ± 0.3	90.6 ± 0.9	85.0 ± 0.8	70.7 ± 0.5

**Table 4:** Removal efficiency of the carbon nanotubes–silica aerogel composites with two amounts of carbon nanostructure for different initial concentrations of xylene.

	Removal Efficiency (%)			
	*C*_0_ = 50 mg⋅L^−1^	*C*_0_ = 100 mg⋅L^−1^	*C*_0_ = 200 mg⋅L^−1^	*C*_0_ = 400 mg⋅L^−1^
100M_CNT-TMOS_10	99.5 ± 0.1	99.7 ± 0.1	96.2 ± 0.4	73.7 ± 2.6
100M_CNT-TMOS_50	98.7 ± 0.3	99.0 ± 0.1	98.9 ± 0.1	76.4 ± 0.3
100M_CNT-HNO_3__10	96.7 ± 0.1	97.4 ± 0.6	97.7 ± 0.2	81.3 ± 1.7
100M_CNT-HNO_3__50	98.2 ± 0.3	99.1 ± 0.2	97.9 ± 0.1	73.0 ± 1.2

**Table 5:** Removal efficiency of the carbon nanotubes–silica aerogel composites with two amounts of carbon nanostructure for different initial concentrations of phenol.

	Removal Efficiency (%)			
	*C*_0_ = 50 mg⋅L^−1^	*C*_0_ = 100 mg⋅L^−1^	*C*_0_ = 200 mg⋅L^−1^	*C*_0_ = 400 mg⋅L^−1^
90M10A_CNT-TMOS_10	32.4 ± 0.9	31.3 ± 2.4	28.2 ± 2.3	17.2 ± 2.2
90M10A_CNT-TMOS_50	30.6 ± 1.0	26.4 ± 0.6	24.2 ± 1.1	21.0 ± 1.1
90M10A_CNT-HNO_3__10	31.9 ± 0.8	29.0 ± 2.7	24.2 ± 2.2	15.6 ± 1.3
90M10A_CNT-HNO_3__50	30.9 ± 0.4	28.3 ± 1.9	23.8 ± 1.2	21.3 ± 0.6

**Table 6:** Removal efficiency of the carbon nanotubes and graphene oxide–silica aerogel composites with two amounts of carbon nanostructure for different initial concentrations of amoxicillin.

	Removal Efficiency (%)			
	*C*_0_ = 10 mg⋅L^−1^	*C*_0_ = 20 mg⋅L^−1^	*C*_0_ = 25 mg⋅L^−1^	*C*_0_ = 50 mg⋅L^−1^
80M20A_CNT-TMOS_10	92.3 ± 2.3	83.9 ± 1.1	82.8 ± 1.1	78.2 ± 0.7
80M20A_CNT-TMOS_50	93.4 ± 0.6	93.8 ± 0.6	92.1 ± 0.5	77.3 ± 0.4
80M20A_GO_10	91.8 ± 1.2	81.6 ± 0.9	79.8 ± 0.3	73.8 ± 1.5
80M20A_GO_50	89.0 ± 4.3	83.6 ± 1.7	80.7 ± 0.3	71.5 ± 1.3

**Table 7:** Removal efficiency of the carbon nanotubes and graphene oxide–silica aerogel composites with two amounts of carbon nanostructure for different initial concentrations of naproxen.

	Removal Efficiency (%)			
	*C*_0_ = 10 mg⋅L^−1^	*C*_0_ = 20 mg⋅L^−1^	*C*_0_ = 25 mg⋅L^−1^	*C*_0_ = 50 mg⋅L^−1^
100M_CNT-TMOS_10	92.4 ± 1.6	89.9 ± 0.3	91.3 ± 0.1	92.1 ± 0.4
100M_CNT-TMOS_50	84.7 ± 0.5	87.5 ± 0.4	89.1 ± 0.8	89.3 ± 0.7
100M_CNT-HNO_3__10	88.2 ± 0.4	93.1 ± 0.2	93.5 ± 0.2	94.8 ± 0.1
100M_CNT-HNO_3__50	88.5 ± 1.1	90.1 ± 0.6	89.6 ± 0.9	91.6 ± 0.6
90M10A_GO_10	94.0 ± 0.6	95.3 ± 0.3	96.2 ± 0.8	98.2 ± 0.4
90M10A_GO_50	97.2 ± 2.9	96.2 ± 0.6	96.4 ± 0.4	98.9 ± 0.1

## Data Availability

Not applicable.

## Supplementary Materials

The following supporting information can be downloaded at: https://www.mdpi.com/article/10.3390/toxics11030232/s1, Table S1: Parameters of non-linear isotherms and kinetic models for benzene adsorption on the silica-based aerogels; Table S2: Parameters of non-linear isotherms and kinetic models for toluene adsorption on the silica-based aerogels; Table S3: Parameters of non-linear isotherms and kinetic models for xylene adsorption on the silica-based aerogels; Table S4: Parameters of non-linear isotherms and kinetic models for phenol adsorption on the silica-based aerogels with carbon nanotubes; Table S5: Parameters of non-linear isotherms and kinetic models for phenol adsorption on silica-based aerogels with graphene oxide; Table S6: Parameters of non-linear isotherms models for amoxicillin adsorption on the 90M10A silica-based aerogels with carbon nanotubes; Table S7: Parameters of non-linear isotherms models for amoxicillin adsorption on the 80M20A silica-based aerogels with carbon nanotubes; Table S8: Parameters of non-linear isotherms models for amoxicillin adsorption on the silica-based aerogels with graphene oxide; Table S9: Parameters of non-linear kinetic models for amoxicillin adsorption on the silica-based aerogels with carbon nanotubes and graphene oxide; Table S10: Parameters of non-linear isotherms and kinetic models for naproxen adsorption on the 100M silica aerogels with carbon nanotubes; Table S11: Parameters of non-linear isotherms and kinetic models for naproxen adsorption on the 90M10A silica aerogels with carbon nanotubes; Table S12: Parameters of non-linear isotherms and kinetic models for naproxen adsorption on the 90M10A silica aerogels with graphene oxide.
