# Supplementary material for: Carbon Nanostructures—Silica Aerogel Composites for Adsorption of Organic Pollutants
Source: Toxics. 2023 Feb 28;11(3):232. doi: 10.3390/toxics11030232 (PMC10059775; doi:10.3390/toxics11030232)
Supplement: Supplementary file 1 [file toxics-11-00232-s001.zip › toxics-2213192-supplementary.pdf]

Supplementary Material

Carbon nanostructures-silica aerogel composites for adsorption of organic pollutants

Alyne Lamy-Mendes [1,2] *, David Lopes [1], Ana V. Girão [2], Rui F. Silva [2] , Wim. J. Malfait [3] and Luísa Durães [1,*]

[1] University of Coimbra, CIEPQPF—Chemical Process Engineering and Forest Products Research Centre, Department of Chemical Engineering, 3030-790 Coimbra, Portugal
[2] CICECO—Aveiro Institute of Materials, Department of Materials and Ceramic Engineering, University of Aveiro, 3810-193 Aveiro, Portugal
[3] Laboratory for Building Energy Materials and Components, Swiss Federal Laboratories for Materials Science and Technology, Überlandstrasse 129, 8600 Dübendorf, Switzerland
* Correspondence: alyne@eq.uc.pt (A.L.-M.); luisa@eq.uc.pt (L.D.)

Table S1. Parameters of non-linear isotherms and kinetic models for benzene adsorption on the silica-based aerogels.

Isotherm Model	Parameters	100M	100M_CNT-TMOS_10	100M_CNT-HNO$_3$_10
Langmuir	q_m (mg.g^{-1})	88.1 ± 5.4	70.7 ± 6.0	78.5 ± 5.8
	K_L (L.mg^{-1})	0.13 ± 0.07	0.12 ± 0.02	0.05 ± 0.01
	R_L	0.017 – 0.43	0.016 – 0.45	0.038 – 0.67
	AIC	42.8	31.8	27.6
Freundlich	$1/n_F$	0.4 ± 0.1	0.3 ± 0.1	0.4 ± 0.1
	K_F ((mg.g^{-1})(L.mg^{-1})$^{1/nF}$)	12.7 ± 2.0	15.3 ± 1.0	7.6 ± 0.7
	AIC	32.7	32.6	29.0
BET	q_S (mg.g^{-1})	62.2 ± 3.8	44.1 ± 4.3	53.7 ± 5.9
	C_{BET} (L.mg^{-1})	135.7 ± 38.2	144.4 ± 18.1	62.8 ± 8.8
	C_S (mg.L^{-1})	587.6 ± 75.3	550.3 ± 77.6	698.2 ± 157.2
	AIC	34.8	27.0	27.0
Maximum experimental q_e (mg.g^{-1})		114.4 ± 2.1	111.4 ± 3.9	103.0 ± 4.8
Removal efficiency (%, C_0 = 200 mg.L^{-1})		72.1 ± 2.0	53.9 ± 2.4	60.0 ± 3.0
**Kinetic Model**	**Parameters**	**100M**	**100M_CNT-TMOS_10**	**100M_CNT-HNO$_3$_10**
Pseudo-first order	k_1 (1.min^{-1})	0.3 ± 0.2	0.3 ± 0.1	0.05 ± 0.01
	q_e (mg.g^{-1})	34.1 ± 0.5	34.7 ± 0.2	30.5 ± 0.6
	AIC	45.5	33.7	35.6
Pseudo-second order	k_e x 10^3 (g.(mg.min^{-1}))	17.3 ± 6.7	30.6 ± 7.2	2.3 ± 0.2
	q_e (mg.g$_{-1}$)	35.1 ± 0.5	35.1 ± 0.2	33.8 ± 0.4
	AIC	40.1	27.5	16.3
Experimental q_e at C_0 of 100 ppm (mg.g^{-1})		41.0 ± 1.6	41.7 ± 1.5	37.7 ± 1.4

Table S2. Parameters of non-linear isotherms and kinetic models for toluene adsorption on the silica-based aerogels.

Isotherm Model	Parameters	100M	100M_CNT-TMOS_10	100M_CNT-HNO$_3$_10
Langmuir	q_m (mg.g^{-1})	206 ± 10.7	306.4 ± 69.4	183.7 ± 11.1
	K_L (L.mg^{-1})	0.02 ± 0.01	0.00 ± 0.01	0.04 ± 0.01
	R_L	0.09 – 0.83	0.22 – 0.93	0.051 – 0.73
	AIC	23.7	45.4	26.7
Freundlich	$1/n_F$	0.47 ± 0.03	0.61 ± 0.06	0.35 ± 0.03
	K_F ((mg.g^{-1})(L.mg^{-1})$^{1/nF}$)	14.2 ± 2.0	7.4 ± 2.1	28.3 ± 4.2
	AIC	27.2	39.9	26.1
BET	q_S (mg.g^{-1})	147.8 ± 23.7	86.3 ± 9.6	123.2 ± 8.3
	C_{BET} (L.mg^{-1})	24.8 ± 6.3	21.7 ± 8.7	49.5 ± 6.5
	C_S (mg.L^{-1})	862.8 ± 421.1	305.3 ± 33.6	547.2 ± 86.0
	AIC	27.9	41.7	20.6
Maximum experimental q_e (mg.g^{-1})		159.6 ± 2.6	169.6 ± 1.3	168.3 ± 2.8
**Kinetic Model**	**Parameters**	**100M**	**100M_CNT-TMOS_10**	**100M_CNT-HNO$_3$_10**
Pseudo-first order	k_1 (1.min^{-1})	0.18 ± 0.11	0.10 ± 0.01	0.078 ± 0.007
	q_e (mg.g^{-1})	44.3 ± 0.4	46.2 ± 0.1	46.3 ± 0.5
	AIC	52.9	36.5	30.0
Pseudo-second order	k_e x 10^3 (g.(mg.min^{-1}))	6.3 ± 0.8	8.6 ± 1.0	6.8 ± 0.8
	q_e (mg.g$_{-1}$)	46.8 ± 0.3	47.2 ± 0.1	47.6 ± 0.3
	AIC	25.4	29.4	16.3
Experimental q_e at C_0 of 100 ppm (mg.g^{-1})		44.3 ± 0.4	43.2 ± 0.8	45.9 ± 0.2

Table S3. Parameters of non-linear isotherms and kinetic models for xylene adsorption on the silica-based aerogels.

Isotherm Model	Parameters	100M	100M_CNT-TMOS_10	100M_CNT-HNO$_3$_10
Langmuir	q_m (mg.g^{-1})	185.9 ± 7.3	190.0 ± 9.2	175.2 ± 6.7
	K_L (L.mg^{-1})	0.12 ± 0.03	0.096 ± 0.026	0.24 ± 0.09
	R_L	0.016 – 0.46	0.020 – 0.51	0.008 – 0.29
	AIC	28.9	32.9	29.2
Freundlich	$1/n_F$	0.2 ± 0.7	0.2 ± 0.1	0.2 ± 0.1
	K_F ((mg.g^{-1})(L.mg^{-1})$^{1/nF}$)	81.5 ± 185.6	64.7 ± 7.7	74.1 ± 12.6
	AIC	91.0	27.2	30.3
BET	q_S (mg.g^{-1})	142.8 ± 5.4	136.8 ± 11.0	142.2 ± 12.5
	C_{BET} (L.mg^{-1})	254.4 ± 75.7	191.9 ± 96.5	272.8 ± 159.9
	C_S (mg.L^{-1})	531.2 ± 70.4	517.7 ± 122.0	456.8 ± 173.1
	AIC	20.8	32.8	29.6
Maximum experimental q_e (mg.g^{-1})		187.3 ± 2.3	184.3 ± 1.8	200.5 ± 4.9
**Kinetic Model**	**Parameters**	**100M**	**100M_CNT-TMOS_10**	**100M_CNT-HNO$_3$_10**
Pseudo-first order	k_1 (1.min^{-1})	0.09 ± 0.01	0.10 ± 0.01	0.10 ± 0.01
	q_e (mg.g^{-1})	48.1 ± 0.2	48.1 ± 0.04	47.8 ± 0.4
	AIC	40.7	22.9	12.5
Pseudo-second order	k_e x 10^3 (g.(mg.min^{-1}))	6.6 ± 0.9	10.4 ± 2.6	3.8 ± 1.1
	q_e (mg.g$_{-1}$)	49.1 ± 0.2	48.7 ± 0.2	50.3 ± 1.1
	AIC	39.0	42.0	24.7
Experimental q_e at C_0 of 100 ppm (mg.g^{-1})		49.9 ± 0.1	49.8 ± 0.1	48.7 ± 0.3

Table S4.Parameters of non-linear isotherms and kinetic models for phenol adsorption on the silica-based aerogels with carbon nanotubes.

Isotherm Model	Parameters	90M10A	90M10A _CNT-TMOS_10	90M10A _CNT-HNO_3_10
Langmuir	q_m (mg.g^{-1})	48.0 ± 9.7	67.9 ± 7.0	53.1 ± 4.0
	K_L (L.mg^{-1})	0.006 ± 0.001	0.004 ± 0.001	0.006 ± 0.001
	R_L	0.26 – 0.95	0.32 – 0.96	0.26 -0.95
	AIC	25.5	9.1	18.1
Freundlich	$1/n_F$	0.75 ± 0.07	0.74 ± 0.03	0.65 ± 0.02
	K_F ((mg.g^{-1})(L.mg^{-1})$^{1/nF}$)	0.52 ± 0.13	0.58 ± 0.07	0.80 ± 0.09
	AIC	38.4	16.5	19.9
Maximum experimental q_e (mg.g^{-1})		32.4 ± 4.8	44.7 ± 5.5	37.9 ± 2.4
**Kinetic Model**	**Parameters**	**90M10A**	**90M10A _CNT-TMOS_10**	**90M10A _CNT-HNO_3_10**
Pseudo-first order	k_1 (1.min^{-1})	0.1 ± 0.3	0.04 ± 0.01	0.25 ± 0.04
	q_e (mg.g^{-1})	14.6 ± 0.2	14.3 ± 0.1	16.2 ± 0.1
	AIC	50.5	52.9	21.0
Pseudo-second order	k_e x 10^3 (g.(mg.min^{-1}))	20.5 ± 3.6	4.9 ± 0.5	47.1 ± 12.4
	q_e (mg.g$_{-1}$)	15.2 ± 0.2	15.1 ± 0.1	16.4 ± 0.1
	AIC	36.0	42.9	19.2
Experimental q_e at C_0 of 100 ppm (mg.g^{-1})		14.5 ± 1.9	15.7 ± 1.4	14.5 ± 1.6

Table S5. Parameters of non-linear isotherms and kinetic models for phenol adsorption on silica-based aerogels with graphene oxide.

Isotherm Model	Parameters	90M10A	90M10A _GO_10
Langmuir	q_m (mg.g^{-1})	23.3 ± 1.4	19.6 ± 1.4
	K_L (L.mg^{-1})	0.011 ± 0.007	0.013 ± 0.001
	R_L	0.16 – 0.95	0.13 – 0.94
	AIC	15.9	27.8
Freundlich	$1/n_F$	0.6 ± 0.1	0.6 ± 0.1
	K_F ((mg.g^{-1})(L.mg^{-1})$^{1/nF}$)	0.5 ± 0.1	0.4 ± 0.1
	AIC	39.0	38.4
Maximum experimental q_e (mg.g^{-1})		19.5 ± 1.6	17.9 ± 0.9
**Kinetic Model**	**Parameters**	**90M10A**	**90M10A _GO_10**
Pseudo-first order	k_1 (1.min^{-1})	0.08 ± 0.01	0.06 ± 0.01
	q_e (mg.g^{-1})	14.2 ± 0.1	10.1 ± 0.1
	AIC	31.5	42.9
Pseudo-second order	k_e x 10^3 (g.(mg.min^{-1}))	12.5 ± 1.6	10.3 ± 0.8
	q_e (mg.g$_{-1}$)	14.6 ± 0.1	10.6 ± 0.1
	AIC	26.1	19.0
Experimental q_e at C_0 of 100 ppm (mg.g^{-1})		11.5 ± 0.5	10.3 ± 0.4

Table S6. Parameters of non-linear isotherms models for amoxicillin adsorption on the 90M10A silica-based aerogels with carbon nanotubes.

Isotherm Model	Parameters	90M10A	90M10A _CNT-TMOS_10	90M10A _CNT-HNO_3_10
Langmuir	q_m (mg.g^{-1})	3.5 ± 0.4	0.8 ± 0.1	6.7 ± 1.5
	K_L (L.mg^{-1})	0.08 ± 0.02	3.4 x10^3 ± 3.1 x10^7	0.06 ± 0.02
	R_L	0.20 – 0.55	0	0.25 – 0.63
	AIC	27.1	34.0	33.1
Freundlich	$1/n_F$	0.38 ± 0.05	0.06 ± 0.01	0.51 ± 0.13
	K_F ((mg.g^{-1})(L.mg^{-1})$^{1/nF}$)	0.7 ± 0.1	0.7 ± 0.3	0.8 ± 0.3
	AIC	24.6	33.7	36.3
Maximum experimental q_e (mg.g^{-1})		2.8 ± 0.2	1.0 ± 0.1	4.6 ± 0.3

Table S7. Parameters of non-linear isotherms models for amoxicillin adsorption on the 80M20A silica-based aerogels with carbon nanotubes.

Isotherm Model	Parameters	80M20A	80M20A _CNT-TMOS_10	80M20A _CNT-HNO_3_10
Langmuir	q_m (mg.g^{-1})	36.0 ± 4.3	45.4 ± 10.6	26.8 ± 2.3
	K_L (L.mg^{-1})	0.09 ± 0.02	0.07 ± 0.03	0.12 ± 0.02
	R_L	0.18 – 0.52	0.22 – 0.59	0.14 – 0.46
	AIC	20.8	22.9	32.5
Freundlich	$1/n_F$	0.63 ± 0.02	0.71 ± 0.07	0.56 ± 0.03
	K_F ((mg.g^{-1})(L.mg^{-1})$^{1/nF}$)	3.9 ± 0.2	3.6 ± 0.5	3.8 ± 0.2
	AIC	14.8	22.4	29.4
Maximum experimental q_e (mg.g^{-1})		18.9 ± 0.1	19.5 ± 0.2	17.4 ± 0.3

Table S8. Parameters of non-linear isotherms models for amoxicillin adsorption on the silica-based aerogels with graphene oxide.

Isotherm Model	Parameters	90M10A	90M10A_GO_10	80M20A	80M20A_GO_10
Langmuir	q_m (mg.g^{-1})	14.7 ± 0.1	11.6 ± 1.9	24.7 ± 2.1	33.5 ± 4.3
	K_L (L.mg^{-1})	0.40 ± 0.02	0.11 ± 0.04	0.20 ± 0.06	0.09 ± 0.02
	R_L	0.048 – 0.20	0.22 – 0.59	0.091 – 0.33	0.18 – 0.53
	AIC	8.8	44.9	25.6	22.9
Freundlich	$1/n_F$	0.21 ± 0.03	0.37 ± 0.04	0.45 ± 0.03	0.61 ± 0.03
	K_F ((mg.g^{-1})(L.mg^{-1})$^{1/nF}$)	6.8 ± 0.6	2.5 ± 0.2	5.8 ± 0.5	3.8 ± 0.3
	AIC	28.5	38.3	20.5	17.5
Maximum experimental q_e (mg.g^{-1})		13.4 ± 0.2	9.3 ± 0.2	18.3 ± 0.2	18.4 ± 0.4

Table S9. Parameters of non-linear kinetic models for amoxicillin adsorption on the silica-based aerogels with carbon nanotubes and graphene oxide.

Kinetic Model	Parameters	80M20A	80M20A _CNT-TMOS_10	80M20A	80M20A_GO_10
Pseudo-first order	k_1 (1.min^{-1})	-2x10^{-7} ± 6x10^5	3x10^{-3} ± 1x10^{-4}	5x10^{-3} ± 7x10^{-4}	6x10^{-3} ± 6x10^{-4}
	q_e (mg.g^{-1})	- 2x10^{-4} ± 8x10^6	9.5 ± 0.2	8.9 ± 0.7	10.5 ± 0.5
	AIC	103.5	34.3	55.2	37.7
Pseudo-second order	k_e x 10^3 (g.(mg.min^{-1}))	0.24 ± 0.05	0.28 ± 0.01	51x10^3 ± 2x10^{10}	0.32 ± 0.09
	q_e (mg.g$_{-1}$)	9.7 ± 0.7	11.5 ± 0.3	2.8 ± 1.0	14.3 ± 1.4
	AIC	54.1	33.7	100.1	44.9
Experimental q_e at C_0 of 25 ppm (mg.g^{-1})		9.7 ± 0.2	9.8 ± 0.1	10.5 ± 0.1	10.0 ± 0.1

Table S10. Parameters of non-linear isotherms and kinetic models for naproxen adsorption on the 100M silica aerogels with carbon nanotubes.

Isotherm Model	Parameters	100M	100M_CNT-TMOS_10	100M_CNT-HNO$_3$_10
Freundlich	1/n_F	1.2 ± 0.1	1.1 ± 0.1	1.8 ± 0.2
	K_F ((mg.g^{-1})(L.mg^{-1})$^{1/nF}$)	2.1 ± 0.2	4.9 ± 0.5	4.4 ± 0.5
	AIC	-2.7	-6.0	-
BET	q_S (mg.g^{-1})	10.7 ± 3.4	11.5 ± 5.4	-
	C_{BET} (L.mg^{-1})	3.2 ± 1.2	3.8 ± 2.3	-
	C_S (mg.L^{-1})	11.2 ± 1.6	6.7 ± 1.5	-
	AIC	21.1	21.4	-
Maximum experimental q_e (mg.g^{-1})		21.7 ± 0.1	23.0 ± 0.1	23.7 ± 0.1
**Kinetic Model**	**Parameters**	**100M**	**100M_CNT-TMOS_10**	**100M_CNT-HNO$_3$_10**
Pseudo-first order	k_1 (1.min^{-1})	0.024 ± 0.004	0.014 ± 0.004	0.056 ± 0.005
	q_e (mg.g^{-1})	9.5 ± 0.5	10.5 ± 1.6	9.9 ± 0.2
	AIC	21.8	23.9	20.0
Pseudo-second order	k_e x 10^3 (g.(mg.min^{-1}))	1.4 ± 0.5	0.6 ± 0.3	6.5 ± 0.9
	q_e (mg.g$_{-1}$)	13.5 ± 1.4	16.3 ± 3.5	11.6 ± 0.3
	AIC	23.0	24.4	14.6
Experimental q_e at C_0 of 25 ppm (mg.g^{-1})		10.5 ± 0.1	11.4 ± 0.1	11.7 ± 0.1

Table S11. Parameters of non-linear isotherms and kinetic models for naproxen adsorption on the 90M10A silica aerogels with carbon nanotubes.

Isotherm Model	Parameters	90M10A	90M10A _CNT-TMOS_10	90M10A _CNT-HNO$_3$_10
Langmuir	q_m (mg.g^{-1})	12.5 ± 0.8	12.8 ± 4.4	14.3 ± 1.3
	K_L (L.mg^{-1})	0.8 ± 0.1	0.05 ± 0.03	0.4 ± 0.1
	R_L	0.024 – 0.11	0.29 – 0.67	0.048 – 0.20
	AIC	-4.9	32.4	12.6
Freundlich	1/n_F	0.16 ± 0.03	0.53 ± 0.30	0.23 ± 0.08
	K_F ((mg.g^{-1})(L.mg^{-1})$^{1/nF}$)	7.4 ± 0.4	1.3 ± 0.3	6.7 ± 1.0
	AIC	10.7	26.5	20.8
BET	q_S (mg.g^{-1})	11.6 ± 0.2	3.7 ± 0.3	-3.5x10^{-5} ± 41.1
	C_{BET} (L.mg^{-1})	373.8 ± 54.9	-6.5x10^7 ± 3.2x10^{12}	0.9 ± 4.2x10^3
	C_S (mg.L^{-1})	381.2 ± 75.1	48.9 ± 7.9	0.3 ± 1.3x10^4
	AIC	12.9	60.4	64.4
Maximum experimental q_e (mg.g^{-1})		12.0 ± 0.9	9.3 ± 0.3	12.4 ± 1.3
**Kinetic Model**	**Parameters**	**90M10A**	**90M10A _CNT-TMOS_10**	**90M10A _CNT-HNO$_3$_10**
Pseudo-first order	k_1 (1.min^{-1})	0.08 ± 0.01	0.1 ± 0.01	0.08 ± 0.01
	q_e (mg.g^{-1})	11.5 ± 0.1	11.3 ± 0.1	12.2 ± 0.1
	AIC	22.0	20.4	22.4
Pseudo-second order	k_e x 10^3 (g.(mg.min^{-1}))	10.0 ± 0.3	19.5 ± 5.6	19.9 ± 4.2
	q_e (mg.g$_{-1}$)	12.9 ± 0.3	12.0 ± 0.2	12.8 ± 0.1
	AIC	20.0	32.2	34.2
Experimental q_e at C_0 of 25 ppm (mg.g^{-1})		9.9 ± 0.2	5.4 ± 0.1	10.1 ± 0.1

Table S12. Parameters of non-linear isotherms and kinetic models for naproxen adsorption on the 90M10A silica aerogels with graphene oxide.

Isotherm Model	Parameters	90M10A	90M10A _GO_10
Langmuir	q_m (mg.g^{-1})	48.5 ± 8.8	-
	K_L (L.mg^{-1})	0.15 ± 0.05	-
	R_L	0.12 – 0.40	-
	AIC	16.4	-
Freundlich	1/n_F	0.72 ± 0.08	2.9 ± 0.6
	K_F ((mg.g^{-1})(L.mg^{-1})$^{1/nF}$)	6.3 ± 0.8	17.6 ± 2.3
	AIC	19.8	-
Maximum experimental q_e (mg.g^{-1})		22.2 ± 0.4	24.5 ± 0.1
**Kinetic Model**	**Parameters**	**90M10A**	**90M10A _GO_10**
Pseudo-first order	k_1 (1.min^{-1})	0.034 ± 0.001	0.04 ± 0.01
	q_e (mg.g^{-1})	11.7 ± 0.1	12.1 ± 0.1
	AIC	22.3	21.7
Pseudo-second order	k_e x 10^3 (g.(mg.min^{-1}))	4.2 ± 0.4	4.5 ± 0.6
	q_e (mg.g$_{-1}$)	12.9 ± 0.2	12.9 ± 0.1
	AIC	30.7	17.4
Experimental q_e at C_0 of 25 ppm (mg.g^{-1})		10.1 ± 0.1	12.0 ± 0.1
